# Broad repression of DNA repair genes in senescent cells identified by integration of transcriptomic data

**DOI:** 10.1093/nar/gkae1257

**Published:** 2024-12-31

**Authors:** Yann Frey, Majd Haj, Yael Ziv, Ran Elkon, Yosef Shiloh

**Affiliations:** The David and Inez Myers Laboratory for Cancer Research, Tel Aviv University, Tel Aviv 6997801, Israel; Department of Human Molecular Genetics and Biochemistry, School of Medicine, Faculty of Medical & Health Sciences, Tel Aviv University, Tel Aviv 6997801, Israel; The David and Inez Myers Laboratory for Cancer Research, Tel Aviv University, Tel Aviv 6997801, Israel; Department of Human Molecular Genetics and Biochemistry, School of Medicine, Faculty of Medical & Health Sciences, Tel Aviv University, Tel Aviv 6997801, Israel; The David and Inez Myers Laboratory for Cancer Research, Tel Aviv University, Tel Aviv 6997801, Israel; Department of Human Molecular Genetics and Biochemistry, School of Medicine, Faculty of Medical & Health Sciences, Tel Aviv University, Tel Aviv 6997801, Israel; Department of Human Molecular Genetics and Biochemistry, School of Medicine, Faculty of Medical & Health Sciences, Tel Aviv University, Tel Aviv 6997801, Israel; The David and Inez Myers Laboratory for Cancer Research, Tel Aviv University, Tel Aviv 6997801, Israel; Department of Human Molecular Genetics and Biochemistry, School of Medicine, Faculty of Medical & Health Sciences, Tel Aviv University, Tel Aviv 6997801, Israel

## Abstract

Cellular senescence plays a significant role in tissue aging. Senescent cells, which resist apoptosis while remaining metabolically active, generate endogenous DNA-damaging agents, primarily reactive oxygen species. Efficient DNA repair is therefore crucial in these cells, especially when they undergo senescence escape, resuming DNA replication and cellular proliferation. To investigate whether senescent cell transcriptomes reflect adequate DNA repair capacity, we conducted a comprehensive meta-analysis of 60 transcriptomic datasets comparing senescent to proliferating cells. Our analysis revealed a striking downregulation of genes encoding essential components across DNA repair pathways in senescent cells. This includes pathways active in different cell cycle phases such as nucleotide excision repair, base excision repair, nonhomologous end joining and homologous recombination repair of double-strand breaks, mismatch repair and interstrand crosslink repair. The downregulation observed suggests a significant accumulation of DNA lesions. Experimental monitoring of DNA repair readouts in cells that underwent radiation-induced senescence supported this conclusion. This phenomenon was consistent across various senescence triggers and was also observed in primary cell lines from aging individuals. These findings highlight the potential of senescent cells as ‘ticking bombs’ in aging-related diseases and tumors recurring following therapy-induced senescence.

## Graphical Abstract

**Graphical Abstract:**
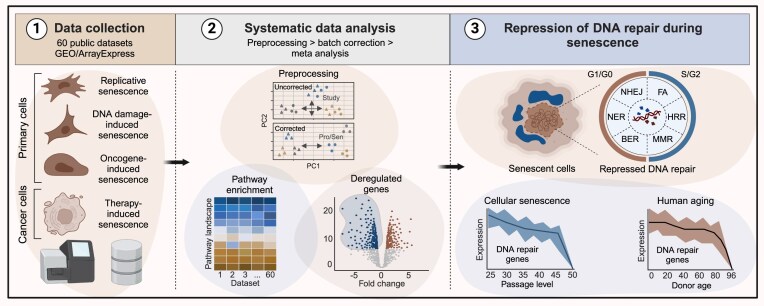

## Introduction

The stability and integrity of cellular DNA are constantly challenged by both spontaneous alterations in DNA components (1) and exposure to DNA-damaging agents (2). Most of these agents are endogenous reactive oxygen species (ROS) generated during normal cellular metabolism, with additional contributions from environmental chemicals or various forms of radiation. By-products from food processing and health-related behaviors such as smoking further increase the DNA damage burden in human tissues. Collectively, it is estimated that tens of thousands of DNA lesions are induced daily in a single cell (3,4). Preserving genome stability against this persistent DNA damage is paramount to preventing unwarranted cell death or the onset of neoplasia (2,5). The primary guardian of genome stability is the DNA damage response (DDR)—an elaborate signal transduction network orchestrating DNA repair pathways while finely modulating numerous physiological processes in a synchronized manner (4,6,7). Mutations compromising DDR function are commonly associated with disorders characterized by progressive degeneration, increased cancer susceptibility and segmental premature aging (8,9). The link between genome instability and accelerated aging is further supported by the accumulation of excessive DNA damage in aging tissues (9,10).

Another prevalent feature of aging tissues is the accumulation of senescent cells (11–14). Cellular senescence is a state marked by prolonged cell cycle arrest, resistance to apoptosis, substantial alterations in cellular morphology, metabolic shifts, chromatin reorganization, and epigenetic and gene expression modifications, along with elevated DNA damage (15–18). A key characteristic of senescent cells is the senescence-associated secretory phenotype (SASP), which involves extensive secretion of cytokines and growth factors, many of which are pro-inflammatory. The SASP can exert autocrine and paracrine effects, profoundly influencing the tissue microenvironment (19,20).

Replicative senescence (RS), the first documented form of cellular senescence, is readily observed in primary fibroblast lines as they undergo successive population doublings, coinciding with a gradual telomere erosion (21). Beyond telomere shortening, various stresses can trigger cellular senescence—a process termed ‘stress-induced premature senescence’ (SIPS). Key examples include DNA damage from oxidative or genotoxic stress, oncogene activation and other cellular insults (15,22,23).

Cellular senescence is an essential but Janus-faced process: on the one hand, it plays essential roles in embryonic development, tissue repair, elimination of damaged cells and tumor suppression. On the other hand, over time, it can drive pathological degeneration, promote inflammatory processes (24), contribute to cancer progression and exacerbate age-related diseases (23,25,26). Recently, growing awareness of the potential reversibility of cellular senescence (24) has underscored the importance of therapy-induced senescence (TIS) and the capacity of senescent cells to escape from cell cycle arrest, which may impact the progression from tumor dormancy to relapse (27–31).

Cellular senescence can be triggered by sustained DNA damage leading to persistent activation of damage-induced cell cycle checkpoints (32). Despite the anticipation that the DDR would mitigate lesion accumulation in senescent cells, these cells often display high levels of ongoing DNA damage (15,16). The underlying causes of this persistent DNA damage remain poorly understood. Continuous accumulation of DNA lesions may impact the metabolism of senescent cells and profoundly destabilize their genome if they re-enter the cell cycle after senescence escape. Thus, the DNA repair capacity of senescent cells is critical not only for normal physiology but also for pathological conditions involving senescence, including TIS, with important clinical implications.

Several reports have noted decreased expression of specific DNA repair genes active during the S and G2 phases of the cell cycle in senescent cultured cells (33–36). These findings prompted us to investigate whether they reflected a broader, programmed suppression of all major DNA repair pathways in senescence. Such suppression, if confirmed, could represent a defining characteristic of senescence with significant biological and clinical implications. To address this question, we performed a comprehensive meta-analysis of 60 publicly available transcriptomic datasets, encompassing both primary cell lines and cancer cell lines. Our analysis revealed widespread downregulation of genes encoding key components across all major DNA repair pathways in senescent cells. This reduction was consistent across various senescence contexts, including replicative, oncogene-induced and DNA damage-induced senescence, as well as senescence induced by treatment with chemotherapeutic agents. Notably, this trend was also observed in datasets with single-cell transcriptomic or proteomic analyses. Importantly, we found that this downregulation intensifies as primary cell lines approach the Hayflick limit. Furthermore, the suppression of DNA repair genes is more pronounced in proliferating fibroblast lines derived from older donors, highlighting the persistence of this phenomenon across different cellular contexts and aging processes.

## Materials and methods

### Transcriptomic analysis

A flowchart illustrating the transcriptomic analysis is provided in Supplementary Figure S1.

#### Building a comprehensive human senescence gene expression dataset: from GEO retrieval to preprocessing pipelines

**Figure 1.**
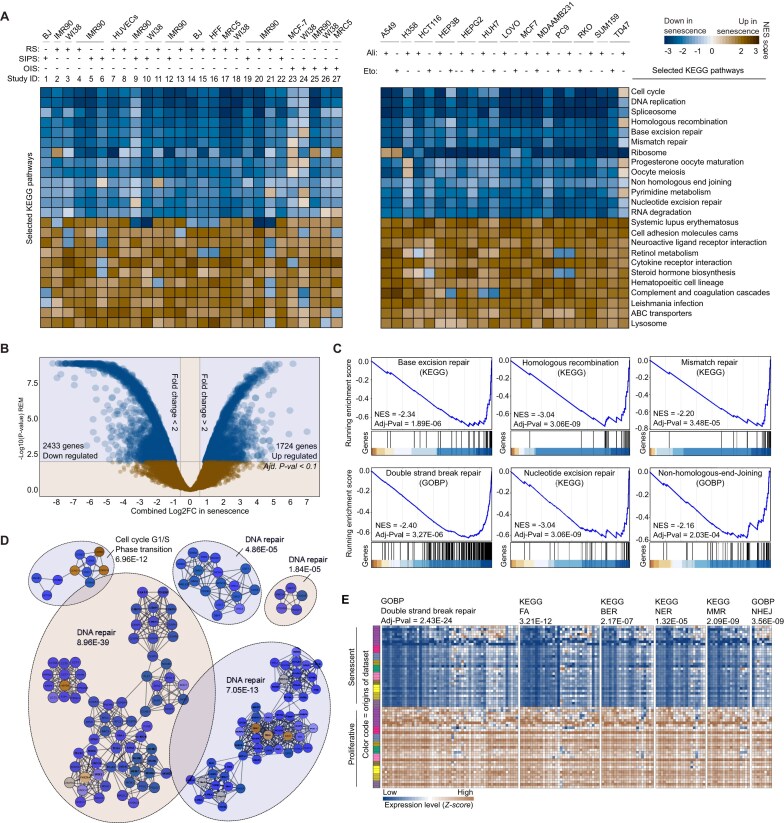
Widespread suppression of DNA repair pathways during senescence. (**A**) Heatmaps of GSEA (gene set enrichment analysis) scores (normalized enrichment score, NES): left panel—GSEA scores for significantly enriched KEGG (Kyoto Encyclopedia of Genes and Genomes) pathways across 27 datasets comparing senescent versus proliferating primary cells, with senescence triggers indicated above the heatmap. Right panel—GSEA scores from the SENESCopedia dataset comparing chemotherapy-induced senescent cells to proliferating controls. Only pathways with a significant average adjusted *P*-value across all studies are shown, except for the NHEJ (nonhomologous end joining) pathway. RS: replicative senescence; SISP: stress-induced premature senescence; OIS: oncogene-induced senescence; Ali: alisertib; Eto: etoposide. (**B**) Volcano plot of meta-analysis results: each dot represents a gene, with the *X*-axis showing the meta-fold change during senescence and the *Y*-axis indicating the −log_2_ adjusted *P*-value from the random effects model (REM). (**C**) GSEA of DNA repair genes in senescent cells: ranked enrichment analysis based on transcriptomic meta-analysis, highlighting negative enrichment of DNA repair genes in senescent cells. (**D**) Topological enrichment of differentially expressed genes (DEGs): protein–protein interaction (PPI) modules constructed using STRING. Blue nodes represent proteins encoded by downregulated genes, while yellow nodes represent those produced by upregulated genes. The functions of these modules were identified via ORA (overrepresentation analysis) enrichment analysis, indicating highly interconnected, predominantly downregulated modules related to DNA repair. (**E**) Concurrent downregulation of DNA repair pathways: heatmaps showing the expression of significantly downregulated DNA repair pathway genes in senescent cells [adjusted *P*-value <0.01; log_2_ fold change (log_2_FC) < −0.58], with study origins color coded in the right panel. Adjusted *P*-values from ORA pathway enrichment tests are displayed above each heatmap.

This section corresponds to Figure 1A. Relevant transcriptomic data for this study were manually curated from PubMed and the Gene Expression Omnibus (GEO) database. We used a dual search strategy, combining database keyword queries (e.g. ‘Senescence’) with a literature review, to identify senescence-related RNA sequencing (RNA-seq) datasets. Literature review tools such as Research Rabbit (https://www.researchrabbit.ai/) and Litmaps (www.litmaps.com) facilitated this process. To control for cell type variability, we initially focused on data derived from two primary cell types: fibroblasts obtained from lung and skin biopsies and endothelial cells (Figure 1A). Subsequently, we gathered and re-analyzed data from the SENESCopedia database (37), allowing us to extend our observation to 13 additional cell lines, including A549, H358, HCT116, HEP3B, HEPG2, HUH7, LOVO, MCF-7, MDA-AMB-231, PC9, RKO, SUM159 and TD47, covering lung adenocarcinoma, colon carcinoma, hepatocellular carcinoma, breast adenocarcinoma, non-small cell lung carcinoma, mesenchymal triple-negative breast cancer and thyroid carcinoma. To capture a wide range of senescence triggers, we selected datasets representing various senescence types, including replicative, DNA damage-induced, therapy-induced and oncogene-induced senescence (OIS). Preliminary dataset selection was based on two main criteria: availability of raw FASTQ/FASTA data for unbiased analysis and inclusion of both proliferative and senescent conditions, each with at least two replicates. Preference was given to datasets with experimental validation.

Raw data were acquired from the Short Read Archive and systematically preprocessed using the Galaxy platform (38). Quality control included initial assessment with FASTQC, followed by read trimming (removal of 10 bases from external regions and reads with a quality score below Q25) using Trimmomatic (39) and FASTQC re-evaluation. Trimmed reads were aligned to the GRCh38/hg38 human genome reference using HISAT2 (40), and alignment quality was verified with MultiQC (41). Gene expression quantification for protein-coding genes (GENCODE V44) was conducted with FeatureCounts (42). To normalize count tables, we applied log counts per million (logCPM) using EdgeRCount (43), utilizing the function calcNormFactors followed by cpm(log = TRUE). Finally, the normalized data were used to validate clear separation between proliferative and senescent conditions through principal component analysis (PCA; R v3.5.3 ‘stats’ package; data not shown).

#### Differential expression analysis

Differential gene expression was performed using the DESeq2 package (44), applying significance thresholds of adjusted *P* < 0.05 and an absolute log_2_FC > 0.58.

#### Assessing gene deregulation consistency across studies

To identify genes consistently deregulated across the analyzed datasets, we assigned a score to each gene based on its differential expression in individual studies. Genes received a score of +1 for upregulation and −1 for downregulation in studies where their expression was significantly altered. For significance thresholds, refer to the ‘Differential expression analysis’ section. Finally, the distribution of gene score was plotted to provide a visual representation of this scoring system (Supplementary Figure S2A).

#### Gene set enrichment analysis

GSEA was conducted using the clusterProfiler package (37). Gene sets from both the KEGG (45) and Gene Ontology Biological Process (GOBP) (46) were used as reference resources. Genes were ranked according to their differential expression between proliferative and senescent (or quiescent) conditions, with fold changes calculated using DESeq2. To visualize the results, GSEA heatmaps were generated (Figure 1A and B, and Supplementary Figure S2B and D), displaying the NES with the Pheatmap package.

#### Enrichment analysis using the ORA

To functionally annotate the DEGs, we conducted Gene Ontology (GO) enrichment analysis using an ORA framework, implemented with the clusterProfiler (37) or EnrichR (47) packages. The background gene set consisted of all protein-coding genes. A significance threshold of adjusted *P*-value <0.05 was applied, using the Benjamini and Hochberg correction for multiple testing. An expression heatmap was generated with NetworkAnalyst (48).

#### Batch correction and REM meta-analysis for identifying robust senescence signatures

To construct a representative super-dataset, we arbitrarily selected 10 datasets from our initial collection based on their distinct separation observed in PCA, using the R ‘stats’ package (Supplementary Figure S3A). Including >10 datasets did not substantially increase the number of identified DEGs and could complicate downstream analyses, as some tools have limitations on the number of samples. The chosen datasets were subjected to logCPM normalization using EdgeR (43) and batch effect correction using ComBAT-seq (49), where the study of origin was used as the batch variable. The comBAT correction was applied using the ‘sva_3.50.0’ R package with Combat()’s default parameters or through the NetworkAnalyst platform (48) with its default setting. Post-correction PCA was conducted to confirm the effective removal of batch effects (Supplementary Figure S3B).

To identify DEGs, we employed an REM meta-analysis, which accounts for heterogeneity among the individual studies. REMs enhance statistical power and robustness, providing more accurate estimates of the overall effect size for DEGs. This analysis was performed using the NetworkAnalyst tool (48) with default settings. We visualized the DEGs using a volcano plot generated with ggplot2. The ‘CombinedES’ REM effect size, which indicates the direction and magnitude of differential expression, and corresponding *P*-values, which reflect the stability of differential expression, were plotted (Figure 1B). Additionally, the ‘CombinedES’ effect size was used to rank genes and conduct a GSEA (Figure 1C), as described earlier. Genes identified as significantly deregulated (*P*-value <0.05 and CombinedES > |0.58|) in the REM analysis underwent ORA using clusterProfiler (37) (Figure 1E, upper values). The corrected count tables from the selected datasets were combined to generate a heatmap displaying our genes of interest (Figure 1E).

#### Gene expression clustering to identify DNA repair repression dynamics

**Figure 2.**
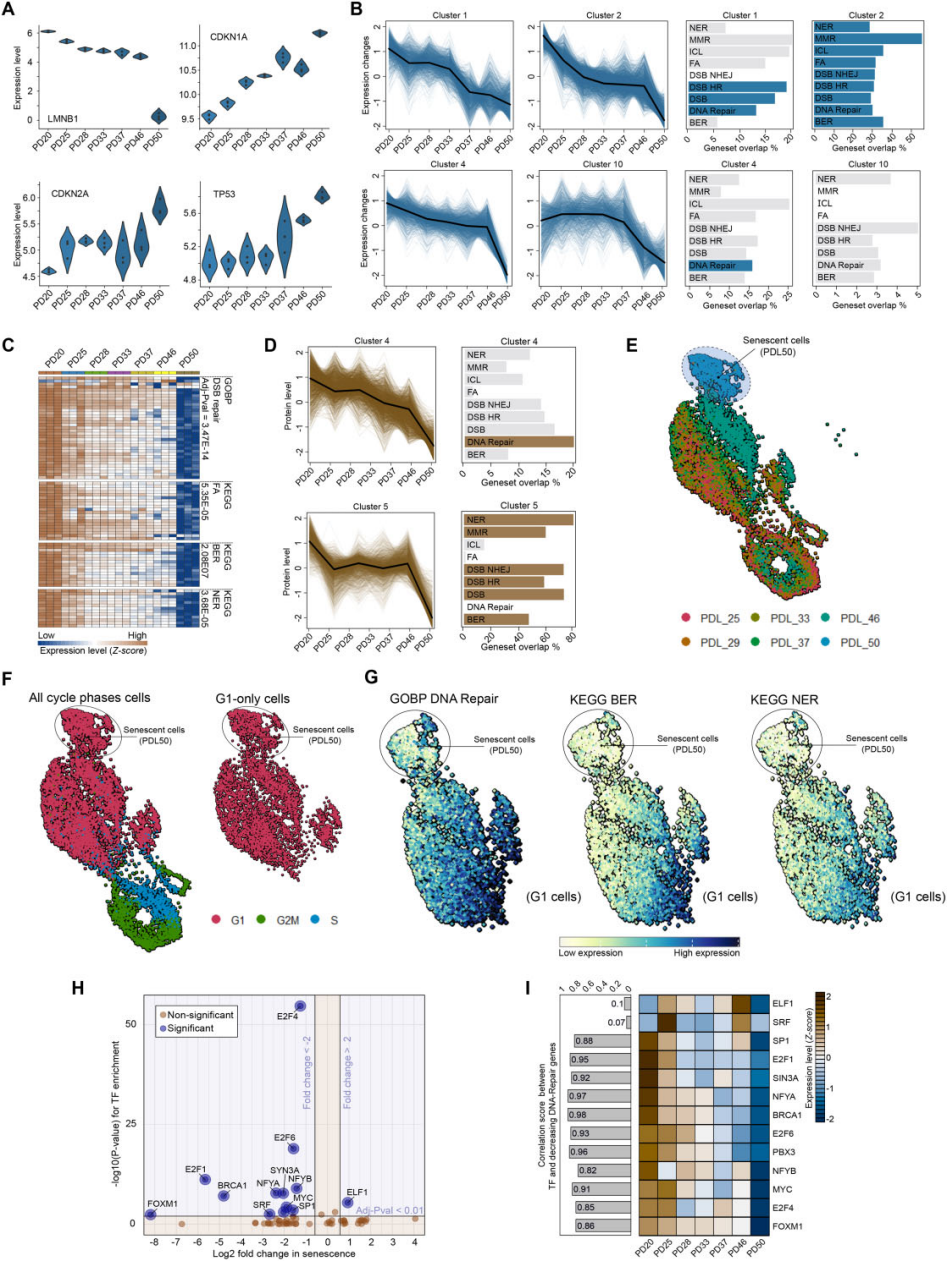
Progressive decline in DNA repair during RS. (**A**) Expression of senescence markers across cell passages, with raw data sourced from GEO (accession number GSE175533). (**B**) Gene expression clusters and pathway enrichment: left panel—four distinct patterns of gene expression decline identified in cells undergoing RS. Right panel—KEGG enrichment plots for DNA repair pathways, highlighting significantly enriched pathways (adjusted *P*-value <0.01) highlighted in blue. (**C**) Heatmaps depicting significantly downregulated DNA repair genes (adjusted *P*-value <0.01, log_2_FC < −0.58) at PD50 compared to PD20. The passage levels are indicated above, and pathway enrichment statistics for the downregulated genes are provided adjacent to each heatmap section. (**D**) Protein level clustering and pathway enrichment analysis: left panel—two distinct clusters of proteins showing progressive decline across cell passages (cluster 4) or a sudden, sharp decline toward PD50 (cluster 5). Right panel—enrichment analysis highlighting DNA repair pathways within these clusters, with significantly enriched pathways (adjusted *P*-value <0.01) marked in brown. Data adapted from (59). (**E**) UMAP projections of single-cell RNA sequencing (scRNA-seq) data by passage level of WI-38 cells (scRNA-seq data: GEO accession number GSE175533), colored according to passage level (population doubling level, PDL). (**F**) UMAP projections of scRNA-seq data colored by cell cycle phase, with the right panel focusing on subclustered G1-phase cells. (**G**) UMAP projections illustrating expression levels of downregulated DNA repair pathways, colored by expression intensity. (**H**) Volcano plot showing transcription factor (TF) gene expression changes during senescence (*X*-axis) and binding enrichment of downregulated DNA repair genes (*Y*-axis). TFs with significant deregulation and binding sites in regulatory regions of downregulated DNA repair genes are labeled in blue. (**I**) Heatmap of TF expression changes across passage levels, highlighting TFs identified in panel (H). The bar chart on the left displays correlation scores between TF expression and median DNA repair gene expression across passage levels.

Gene expression clustering was performed for both cell passage (Figure 2B) and aging analyses (Figure 4A) using the Mfuzz software (50). The optimal number of clusters was determined with the RNfuzzyApp (51) utilizing its built-in elbow method. Subsequently, the identified clusters were used to identify pathway enrichment as previously described. A similar approach was applied to cluster protein level across passage (Figure 2D). In brief, protein level data were directly obtained from the authors’ repository (https://github.com/dghendrickson/hayflick/tree/main/data/proteomics) and log_2_FC values were used as input for the Mfuzz software (50).

#### Identification of TFs involved in gene repression

To identify TFs potentially responsible for the repression of DNA repair genes, we compiled a list of 159 downregulated repair genes. An overlap analysis was conducted using the EnrichR tool (47) and its ‘ENCODE and ChEA Consensus TFs from ChIP-X’ database to detect TFs with binding motifs that were statistically overrepresented in our gene set.

#### scRNA-seq analysis

Data were obtained from the GEO database (accession ID: GSE175533). The H5AD data were downloaded and converted into Seurat object using the SCEasy Converter (52) The Seurat object was then loaded in R, and classical quality control, QCmetric filtering, was applied (53) using Seurat platform (54). The data were normalized using the SCtransform method (55) as part of the Seurat workflow. Cell cycle phases were annotated with the CellCycleScoring function, and cells were projected using UMAP. Clustering was validated using the Louvain algorithm and compared with PDL and cell cycle phase.

DEGs between clusters were identified using the FindMarkers function, with their fold change values subsequently used for GSEA to identify enriched pathways. To visualize downregulated pathways of interest on the UMAP, Seurat modules were created using the AddModuleScore function, and Scpubr (56) was employed for visualization.

### Experimental procedures

#### Cell culture and treatment

CAL-51 cells were maintained in complete Dulbecco’s modified Eagle medium supplemented with 10% fetal bovine serum at 37°C, 5% CO_2_ and ambient oxygen. Senescence was induced by exposing the cultures to 10 Gy ionizing radiation (IR) in an RX-650 irradiation cabinet (Faxitron Bioptics, LLC, Tucson, AZ, USA), followed by incubation for 10 days. To induce DNA damage, cells were exposed to 0.5 Gy IR, treated with 5 mM KBrO_3_ (Sigma–Aldrich, St Louis, MO, USA) for 9 h followed by two phosphate-buffered saline (PBS) washes, or subjected to 30 J/m^2^ UVC radiation using a Stratalinker UV 2400 (Agilent Technologies, Inc., Santa Clara, CA, USA) after medium removal and washing to optimize exposure.

#### Fluorescent assays

Senescence-associated β-galactosidase (SA-β-Gal) imaging was performed as previously described (49). For immunofluorescent detection of DNA lesions or associated markers, 5 × 10^4^ cells per well were seeded in 24-well plates with coverslips precoated with 0.2% gelatin (Sigma–Aldrich). Twenty-four hours post-seeding, cells were exposed to genotoxic agents, washed twice with PBS and fixed in 4% paraformaldehyde for 20 min at room temperature (RT). Cells were permeabilized with 0.5% Triton X-100 (Sigma–Aldrich) for 15 min, followed by two PBS washes. For cyclobutane pyrimidine dimer (CPD) staining, DNA was denatured *in situ* using 2 M HCl for 30 min at RT. Blocking was performed in a buffer containing PBS, 5% bovine serum albumin and 10% normal donkey serum, followed by incubation with primary antibodies for 1.5 h at RT with gentle agitation. Primary antibodies included anti-53BP1 (1:500, Novus Biologicals, Centennial, CO, USA), anti-γH2AX (1:500, Sigma–Aldrich) and anti-CPD (1:10 000, Clone TDM-2, Cosmo Bio Co., Ltd, Tokyo, Japan). Slides were washed three times for 5 min each with PBS containing 0.1% Triton X-100 and incubated with secondary antibodies (Alexa Fluor 647-conjugated goat anti-rabbit IgG or Alexa Fluor 488-conjugated goat anti-mouse IgG, 1:1000, Thermo Fisher Scientific, Waltham, MA, USA) in blocking buffer for 30 min. Final washes (three times, 5 min each) were done with PBS containing 0.1% Triton X-100. Coverslips were mounted on slides using DAPI-containing mounting medium (GBI Labs, Kentwood, MI, USA), dried at RT and stored at 4°C, protected from light. Images of randomly selected fields were captured using a Nikon Eclipse Ti-S inverted microscope (Nikon, Tokyo, Japan). For poly(ADP-ribose) (PAR) chain staining, cells were treated with 10 μM poly(ADP-ribose) glycohydrolase inhibitor (PARGi) for 30 min and fixed in ice-cold acetone:methanol (1:1) for 20 min. After two PBS washes, immunostaining was performed as previously described, with slides incubated with an anti-ADP-ribose reagent (1:800, Millipore–Sigma, Burlington, MA, USA) for 2 h. Following two washes with PBS containing 0.2% Triton X-100, cells were incubated with an Alexa Fluor 647-conjugated goat anti-rabbit IgG secondary antibody (1:1000). Coverslips were mounted with DAPI-containing medium, dried and stored at 4°C. Images were captured as detailed earlier.

## Results

### Repression of genes involved in major DNA repair pathways during senescence

Supplementary Table S1 provides a list of publications representing the datasets used in this study (57–76). The transcriptomic landscape of cellular senescence varies considerably based on cell type, senescence trigger and microenvironmental context (15,62). To enhance the statistical power of our analysis, we focused on three primary cell types: lung fibroblasts, skin fibroblasts and endothelial cells. To cover a broad spectrum of senescence triggers, we included RS, OIS, SIPS caused by DNA-damaging agents and chemotherapeutic drugs known to cause TIS. Furthermore, we ensured that the selected datasets contained validated senescence markers, such as proliferation arrest, SA-β-Gal activity and changes in key protein levels such as p21, p16 or LAMIN B1.

To enable cross-study comparisons and minimize potential biases arising from the varying preprocessing methods used by different investigators, we conducted our own preprocessing of the raw data (Supplementary Figure S1). Dataset quality was evaluated by examining the distinct separation between the transcriptomes of proliferating and senescent cells using PCA. Following preprocessing, each dataset was individually analyzed to identify DEGs (Supplementary Table S2A, individual DEGs). Notably, genes within the ‘KEGG_CELL_CYCLE’ pathway showed significantly reduced expression in senescent cells across all selected datasets, further supporting evidence of senescence induction.

To assess the consistency of gene deregulation during senescence, each gene in the compiled database was assigned a score based on the number of re-analyzed datasets in which its expression was significantly deregulated. A score of +1 was assigned for significant upregulation and −1 for significant downregulation. The distribution of these scores (Supplementary Figure S2A) showed that ∼50% of the genes had scores between −5 and +5, suggesting inconsistent deregulation during senescence. Notably, this distribution skewed toward negative values, indicating a general trend of transcriptome downregulation during senescence.

Among the most consistently downregulated genes (with scores as low as −25), the majority were involved in cell cycle progression, mitosis and DNA repair. In contrast, consistently upregulated genes included well-documented senescence markers and those associated with G1-phase maintenance and autophagy. Interestingly, *CDKN1A*, which encodes the p21 protein, was upregulated in senescent cells in only 21 out of 27 studies, underscoring the absence of universal senescence markers (62). Importantly, genes whose downregulation is recognized as a hallmark of senescence, particularly those involved in cell cycle progression (e.g. *CENPA*, *AURKB*, *KIF18B*), were downregulated in 24 of the 27 studies. For instance, downregulation of *LMNB1*, a documented senescence hallmark, was noted in 23 of the 27 studies. While our study offers the potential to identify novel senescence markers at the gene expression level, our subsequent analysis focused on the downregulation of DNA repair pathways.

We employed GSEA (77) to investigate pathway enrichment within each study individually (Supplementary Table S3A and B). GSEA, as a ranking-based method, allows for the examination of entire transcriptomic profiles, unlike overrepresentation-based methods that depend on gene subsets defined by arbitrary thresholds (78), which can vary across studies. Using the KEGG pathway database (45) as a reference, we uncovered a complex landscape of deregulated processes during senescence, with substantial variability among studies (Figure 1A, Supplementary Figure S2B and Supplementary Table S3A). Nevertheless, common features emerged across the datasets (Figure 1A, left panel), including well-known senescence markers such as increased lysosomal activity and decreased cell cycle processes and replication. RNA splicing was one of the most consistently and strongly repressed processes.

Notably, DNA repair pathways demonstrated consistent downregulation across all studies (Figure 1A, left panel). Supplementary Table S3A provides detailed statistics for the enrichment of pathways identified in our analysis, while Supplementary Table S2A presents the expression levels of individual genes across the 27 studies. These supplementary materials allow readers to examine the data in greater depth and focus on specific pathways of interest.

Genes encoding key components involved in repair processes active during the S and G2 phases, such as homologous recombination repair (HRR) of DNA double-strand breaks (DSBs), mismatch repair (MMR) and interstrand crosslinks (ICL) were predictably downregulated in senescent cells with halted proliferation. However, major repair pathways active throughout the cell cycle, such as base excision repair (BER) and nucleotide excision repair (NER), were also consistently downregulated across all datasets. BER, in particular, plays a crucial role in addressing a significant portion of DNA lesions continuously induced by endogenous ROS (79,80). Additionally, the NHEJ pathway for DSB repair exhibited a widespread decline during senescence in most studies, with some exceptions (Figure 1A and Supplementary Table S3B). Notably, NHEJ is error-prone (81), and its efficiency may come at the cost of introducing mutations. Single-strand break repair (SSBR), another critical pathway that addresses ongoing DNA damage caused by endogenous agents (82,83), did not show a global downregulation in the GSEA (Supplementary Table S3B). However, key genes encoding essential SSBR components, such as PARP1 [a pivotal enzyme in many DNA repair pathways (84)], FEN1, XRCC1, LIG1, TDP1, PNPK and APTX, were downregulated (Supplementary Table S2A, individual DEGs). Collectively, this downregulation is sufficient to impair SSBR effectiveness.

The ribonucleotide excision repair (RER) pathway is notably absent from both the GO and KEGG pathway databases. We conducted a manual assessment of the expression of genes encoding its key components (Supplementary Table S2A, individual DEGs), revealing their downregulation during senescence. These genes include those encoding RNAseH2 subunits, FEN1, EXO1, POLE and POLD. Notably, the RER pathway primarily operates during the S phase, with many of its constituent genes showing peak expression during this phase. Thus, the downregulation of these genes in senescent cells is expected. However, it is important to highlight that, in proliferating cells, the genes encoding RNAseH2 subunits typically maintain constitutive expression throughout the cell cycle (85).

Direct reversal repair is another repair pathway absent from both the GO and KEGG databases. We performed a manual assessment of the expression of its key genes, MGMT and ALKBH2. MGMT was found to be significantly downregulated during senescence in 9 studies and upregulated in 3 out of 22 studies when analyzing data from primary cell lines (Supplementary Table S2A). The ALKBH2 gene was significantly downregulated in 13 studies based on the same primary cell line data (Supplementary Table S2A). Overall, no consistent trend was observed for these genes, suggesting that direct reversal repair is not actively repressed during senescence.

To ensure that the widespread downregulation observed across multiple DNA repair pathways was not simply due to the repression of shared gene subsets, we compared the downregulated gene sets for each pathway using a Venn diagram (Supplementary Figure S2C). This analysis revealed that the vast majority of downregulated repair genes were specific to their respective pathways.

Given the implications of this study for TIS, we focused on data from cancer cell lines treated with chemotherapeutic agents. The SENESCopedia public database, published by R. Bernards and colleagues (63), includes data on 13 cancer cell lines treated with the chemotherapeutic drugs alisertib and etoposide. Using the preprocessed data provided by the authors (as raw data were unavailable), we calculated the fold change before and after senescence induction (Supplementary Table S2B). GSEA revealed consistent patterns across all cell lines and treatments (Figure 1A, right panel, Supplementary Figure S2D, and Supplementary Table S3A and B). Genes involved in DNA repair pathways, including HRR, MMR, BER and NER, were significantly downregulated during senescence induced by these treatments (Figure 1A, right panel).

Although the downregulation of NHEJ genes did not reach statistical significance in every dataset, a consistent trend toward downregulation was observed (Figure 1A and Supplementary Table S3A and B). The re-analysis of SENESCopedia data expanded the scope of DNA repair repression to 13 additional cell lines (A549, H358, HCT116, HEP3B, HEPG2, HUH7, LOVO, MCF-7, MDA-MB-231, PC9, RKO, SUM159 and TD47), which include lung adenocarcinoma, colon carcinoma, hepatocellular carcinoma, breast adenocarcinoma, non-small cell lung carcinoma, mesenchymal triple-negative breast cancer and thyroid carcinoma, in addition to the primary cell lines, such as WI-38, HFF, IMR-90, MRC5, BJ, MCF-7 and HUVECs. Notably, we did not observe any significant influence of cell type or type of senescence on the DNA repair repression (data not shown).

To determine whether the decrease in DNA repair gene expression was specific to the senescence state or a general consequence of cell cycle arrest, we preprocessed and analyzed the publicly available dataset GSE93535 (64), which compared RNA-seq data from senescent and quiescent cells. GSEA revealed a significant reduction in DNA repair gene expression in senescent cells compared to quiescent cells (Supplementary Figure S2E).

Our findings indicate that the repression of DNA repair genes is a distinct characteristic of senescent cells, rather than a general feature associated with prolonged cell cycle arrest. This phenomenon appears consistent across various cell types experiencing replicative, oncogene-induced and DNA damage-induced senescence. Notably, recent studies in *Caenorhabditis elegans* terminally differentiated somatic cells have shown a broad repression of DNA repair genes mediated by the DREAM complex (86). However, while terminally differentiated somatic cells are typically unable to re-enter the cell cycle and are prone to apoptosis, senescent cells exhibit resistance to apoptosis and may potentially escape senescence to initiate DNA replication.

### Dataset integration enhances statistical power, confirming DNA repair repression as a hallmark of senescence

To provide a comprehensive analysis of DNA repair gene repression in senescent cells, we integrated datasets from multiple studies, treating them as biological replicates. This approach enabled the identification of both common patterns and subtle variations across datasets (87). Integrating different datasets required batch correction to mitigate technical variations (88). We applied batch correction using the ComBAT-seq program (49), which allowed us to separate samples by their cellular states (proliferative or senescent) rather than by their originating study (Supplementary Figure S3A and B). To ensure consistency, we selected 10 datasets where PCA demonstrated a clear separation between proliferative and senescent cells, with minimal variation within each state (Supplementary Figure S3B and Supplementary Table S1). We then merged these datasets, treating samples within the proliferative or senescent groups as replicates, and conducted differential expression analysis on the corrected data.

Various methods are available for integrating different transcriptomic datasets (89), including combining *P*-values (e.g. Fisher’s method or Stouffer’s method) or combining effect sizes (e.g. the fixed effect model and REM) (87). We tested these methods and compared their outputs using a Venn diagram (Supplementary Figure S3C). The DEGs identified by these methods were consistent, confirming the robustness of this analysis. However, each method yielded different numbers of deregulated genes in senescent cells, reflecting differences in stringency. For further analysis, we selected the REM approach (Supplementary Table S4), which, due to its higher stringency, identified 4157 significantly deregulated genes during senescence (with thresholds of adjusted *P*-value <0.01 and |log_2_FC| > 0.58) (Figure 1B). Remarkably, the gene encoding FOXM1, a known TF involved in aging and senescence (90,91), showed the most significant decrease in expression in senescent cells (combined log_2_FC = −8.19, adjusted *P*-value = 1.28E−09), followed by the *LMNB1*, which encodes LAMIN B1 (combined log_2_FC = −7.941 and adjusted *P*-value = 1.31E−08). The combination of batch effect correction and REM analysis proved to be an effective strategy for integrating diverse datasets and significantly enhancing our statistical power.

Building on this solid foundation, we proceeded to a more detailed assessment of DNA repair gene expression during senescence. To perform pathway enrichment analysis, we employed three complementary bioinformatic methods: a ranking-based approach (GSEA), an ORA and a topology-based approach to identify modules within PPI networks (92). Using GSEA with KEGG and GOBP references, we found 141 genes linked to DNA repair pathways that were significantly downregulated during senescence (Supplementary Table S4). These pathways included G2–S phase-associated repair mechanisms (HR, MMR and ICL) as well as pathways active throughout the entire cell cycle (NER, BER and NHEJ). The increased statistical power enabled us to highlight significant downregulation of NHEJ, NER (Figure 1C) and telomere maintenance pathways (Supplementary Table S4) during senescence. Similarly, ORA performed on significantly downregulated genes identified through REM analysis (adjusted *P*-value <0.01 and |log_2_FC| > 0.58) (Supplementary Table S4) demonstrated marked enrichment for DNA repair pathways (Figure 1E). To further illustrate these findings, we created expression heatmaps showing the consistent downregulation of DNA repair pathways across 64 experimental samples (Figure 1E). Finally, we applied a topology-based approach to investigate PPIs among the products of deregulated genes. Using the STRING physical interaction database (93), we constructed a PPI map consisting of 4157 proteins encoded by genes significantly up- or downregulated in senescence (not shown). Within this extensive network, we used the MCODE program (94) to identify the most densely interconnected modules. Notably, five of these modules were significantly enriched with DNA repair proteins, with most nodes representing genes downregulated in senescence (Figure 1D).

To enhance our analysis, we utilized the Pathview tool (95), which allowed for a detailed examination of specific proteins within each DNA repair pathway, providing additional biological insights (Supplementary Figure S4). Our findings revealed that most components of these pathways, regardless of their association with specific cell cycle phases, were downregulated during senescence. Notably, while genes encoding ‘sensor’ and ‘transducer’ components of the DDR remained active, those encoding essential repair enzymes, such as DNA ligases, nucleases and topoisomerases, were significantly repressed. This repression may explain the persistent DDR activation observed alongside DNA-SCARS (DNA Segments with Chromatin Alterations Reinforcing Senescence) in senescent cells (96), suggesting an ineffective DNA repair response.

In conclusion, our analysis offers a higher-resolution perspective on gene expression changes during senescence, providing strong evidence for the substantial downregulation of DNA repair pathways. Additionally, our high-resolution approach highlighted the marked repression of genes encoding spliceosome components during senescence (Supplementary Figure S4G).

### Gradual repression of DNA repair genes during RS in primary fibroblasts

To investigate the dynamics of DNA repair gene downregulation during RS, we aimed to determine whether this process occurred gradually as cells approached senescence or as a sudden event at the onset of overt senescence. For this analysis, we used the dataset GSE175533 (59), which comprises RNA-seq data from primary WI-38 cells (human fetal lung fibroblasts). The study began at 20 population doublings (PDL 20) during active proliferation and included samples taken approximately every 5 PDLs until the cells reached full senescence at PDL 50. The onset of senescence was confirmed by a growth plateau at PDL 50 and validated through SA-β-Gal staining and the expression levels of established senescence markers (*CDKN1A*, *CDKN2A*, *NNMT*, *TGFB2* and *GLB1*).

We applied the same preprocessing techniques to the raw data as used in our previous analyses and plotted the expression levels of key senescence markers (Figure 2A) to verify the occurrence and timing of senescence. Notably, we observed a gradual increase in the expression of genes encoding cyclin inhibitors p21 and p16, along with the p53 protein, as well as a marked decline in *LMNB1* expression at PDL 50 (Figure 2A). This expression pattern confirmed that the cells had entered a senescent state, aligning with the conclusions of the original study.

We then conducted clustering analysis to group genes with similar expression dynamics as cells progressed through different passage levels. This analysis resulted in 12 distinct clusters (Supplementary Figure S5A and Supplementary Table S5), 4 of which displayed a clear downward trend (Figure 2B, left panel). To link biological functions to these clusters, we performed ORA (Figure 2B, right panel). Notably, DNA repair pathways were significantly enriched in three of these clusters (clusters 1, 2 and 4), all of which exhibited a gradual decrease in expression, culminating in a sharp decline at the onset of overt senescence. Cluster 2 was particularly prominent, showing significant enrichment across all DNA repair pathways, indicating a tightly regulated downregulation process. In contrast, other clusters with a decreasing pattern that did not include DNA repair genes maintained stable expression through most passage levels, only exhibiting a sharp decrease at the onset of senescence (e.g. cluster 10 in Figure 2B). Additionally, another cluster (Supplementary Figure S5A, cluster 12) also showed a decreasing pattern but was enriched for ‘Response to virus’ and ‘Cytoplasmic translation’ functions (Supplementary Table S5).

We employed an alternative approach to analyze gene expression dynamics by identifying all genes whose expression significantly decreased at PDL 50 (full senescence) compared to PDL 20 (active proliferation). This analysis identified 1740 downregulated genes (adjusted *P* < 0.01 and log_2_FC < −0.58). Enrichment analysis indicated a significant representation of DNA repair genes within this group (Figure 2C, right panel). An expression heatmap (Figure 2C, left panel) further demonstrated the progressive decline in gene expression throughout the passages, culminating in a pronounced drop as the cells entered overt senescence.

To validate the suppression of DNA repair at the protein level, we integrated proteomic data from (59), which were collected in parallel with the dynamic RNA-seq analysis previously mentioned. Expression clustering analysis revealed two clusters with a decreasing trend during senescence (Figure 2D, left panel). Enrichment analysis of these clusters confirmed the downregulation of DNA repair genes, which corresponded with a reduction in their protein products in senescent cells (Figure 2D, right panel). The only exception was the Fanconi anemia (FA) pathway, which did not show significant enrichment within the decreasing clusters. These results indicate a continuous, gradual decline in the expression of DNA repair genes and their protein products as cells progress toward RS, culminating in a marked reduction once senescence is fully established (Figure 5).

To broaden our understanding of the repression dynamics across different cell types and senescence paradigms, we analyzed additional datasets using a similar approach: GSE109700 for RS in IMR-90 cells (Supplementary Figure S5D), GSE175533 for irradiation-induced senescence in WI-38 cells (Supplementary Figure S5E) and GSE108278 for OIS in IMR-90 cells (Supplementary Figure S5F). Overall, these analyses yielded similar results, showing rapid repression of DNA repair pathways following senescence induction. It is worth noting that not all clusters exhibited significant enrichment, particularly for NER and NHEJ, due to the distribution of these genes across multiple expression subclusters. However, differential expression analysis revealed significant downregulation of all major DNA repair pathways (NHEJ, NER, BER, HRR and ICL repair) (Figure 1A and Supplementary Table S2A). The downregulation of NER and NHEJ genes may not have shown a consistent clustering pattern, indicating variability in their expression profiles.

To explore the heterogeneity and dynamics of DNA repair repression in senescent cells, particularly those in the G1 phase, we reanalyzed scRNA-seq data (accession ID: GSE175533) from (59), to identify subpopulations with distinct DNA repair profiles. H5AD files were downloaded and processed using the Seurat package (97). Cells were visualized using UMAP, with color coding validating their separation based on PDL (Figure 2E). Senescent cells (PDL 50) formed a distinct cluster. To evaluate the impact of cell cycle phase on clustering, we color coded cells according to their phase (Figure 2F, left panel), showing that S and G2 cells formed a distinct cluster at the bottom. Given the influence of the cell cycle on DNA repair pathway activity (6,32,98), we focused on G1-phase cells to isolate the effect of passage level and minimize cell cycle-related variability (Figure 2F, right panel). Differential expression analysis between PDL 25 and PDL 50 indicated significant enrichment of downregulated DNA repair genes, confirming their repression (Supplementary Table S8). G1-phase cells were then color coded based on DNA repair pathway activity, revealing a gradual decline in DNA repair gene expression across passages (Figure 2G). Interestingly, within the PDL 50 cluster, a subset of cells maintained high levels of DNA repair gene expression, suggesting that DNA repair repression is heterogeneous among senescent cells and that this variability might point to potential escapees within the population.

### Bioinformatic identification of TFs regulating DNA repair genes repressed during senescence

We investigated the regulatory regions of downregulated DNA repair genes during senescence to identify specific TF target motifs, including those that may also be downregulated. A volcano plot was created to display TF binding site enrichment versus the fold change in expression of the corresponding genes during senescence (Figure 2H). This analysis revealed 13 out of 69 TFs as potential regulators of various subsets of DNA repair genes (Supplementary Table S6). To further identify TFs most strongly associated with the regulation of DNA repair genes, we analyzed their expression patterns across different passage levels using the GSE175533 dataset (Figure 2I). Notably, TFs E2F1, E2F4, E2F6 and FOXM1 exhibited the strongest correlation with the downregulation of DNA repair genes during RS (Figure 2I, left panel), suggesting their potential role as repressors of these genes in this context. These TFs are well-established regulators of the cell cycle (98–100) and have known associations with DNA repair processes cycle regulators (90,98,101–104). Additionally, NFYA, NFYB and MYC, which are involved in the regulation of other biological processes, also emerged as potential upstream regulators.

These findings underscore the significance of specific TFs whose suppression may contribute to the observed downregulation of DNA repair genes during RS. Given prior evidence implicating the DREAM complex in repressing DNA repair genes in terminally differentiated *C. elegans* cells (86), we performed GSEA to examine the expression of DREAM target genes in our datasets. Notably, these target genes were downregulated (Supplementary Figure S5B and C), suggesting that the DREAM complex may similarly act as a repressor in the context of senescence. Additionally, FOXM1, which has been reported to counteract the repressive activity of the DREAM complex (105), was also found to be downregulated during senescence. This downregulation could potentially enhance DREAM-mediated suppression of DNA repair genes.

### Experimental validation of impaired DNA repair during cellular senescence

**Figure 3.**
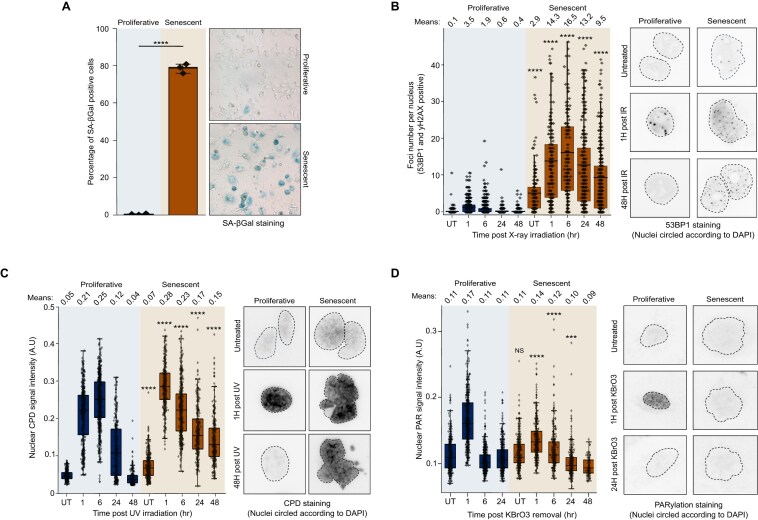
Experimental validation of DNA repair repression during senescence. (**A**) SA-β-Gal staining for quantifying senescence induction. CAL-51 cells were exposed to 10 Gy of X-ray radiation and incubated for 10 days before senescence was quantified. (**B**) Quantification of DSB dynamics following exposure to 0.5 Gy of X-ray radiation. DSB foci were quantified based on the overlap of 53BP1 and γH2AX signals at the indicated time points. (**C**) Quantification of CPD recovery following UV exposure (30 J/m^2^). (**D**) Quantification of poly(ADP-ribosyl)ation (PARylation) dynamics following potassium bromate (KBrO_3_) treatment. Cells were treated with 5 mM KBrO_3_ for 9 h, followed by removal of the treatment. At the indicated time points, cells were treated with a PARGi to enhance PARylation detection before fixation and staining. Statistical significance between proliferative and senescent cells at the same time points was determined using a two-sample Student’s t-test with Welch’s correction: *P < 0.05, **P < 0.01, ***P < 0.001 and ****P < 0.0001.

To validate the repression of DNA repair in senescent cells, we investigated DNA repair dynamics in proliferating and senescent CAL-51 cells exposed to various genotoxic agents that activate distinct repair pathways. Senescence was induced by subjecting the cells to 10 Gy X-ray irradiation, followed by a 10-day culture period, and confirmed through SA-β-Gal staining (Figure 3A). To evaluate DSB repair efficiency, we exposed cells to 0.5 Gy X-rays and monitored the dynamics of co-localized 53BP1 and γH2AX nuclear foci—a well-established marker of DSBs. As expected, senescent cells exhibited elevated basal level of such foci—a known senescence marker (Figure 3B). Notably, both the rapid and slow phases of DSB repair were significantly impaired in senescent cells (Figure 3B).

To assess NER efficiency, we treated cells with 30 J/m^2^ of UVC radiation and monitored the dynamics of CPDs, a primary type of DNA lesion induced by UV exposure, using immunofluorescence. The results demonstrated that senescent cells exhibited a significantly delayed reduction in CPDs over time compared to proliferating cells, indicating impaired NER function in senescent cells (Figure 3C).

As a general indicator of the DDR, we measured PARylation levels, which reflect PARP1 activity, following exposure to KBrO_3_, an oxidative stress and SSB inducer. PARP1 is a key player in multiple DNA repair pathways, including DSB, NER and BER (84). Our transcriptomic meta-analysis identified PARP1 as one of the most downregulated repair factors (Supplementary Figure S2A and Supplementary Table S2A). In proliferating cells, exposure to KBrO_3_ triggered a sharp, transient increase in PARylation, signaling an active repair response to oxidative damage (Figure 3D). In contrast, senescent cells exhibited only a modest increase in PARylation, suggesting impaired or residual PARP1 activity and an overall weakened DDR.

These findings confirm a broad impairment in DNA repair capacity in senescent cells, consistent with the transcriptional repression of DNA repair pathways observed in our computational analyses. The results underscore the heightened vulnerability of senescent cells to DNA damage and their significantly compromised repair mechanisms.

### Shared repression of DNA repair genes in senescing cells and proliferating fibroblast lines from aging individuals

We explored whether the repression of DNA repair genes is a characteristic feature of proliferating dermal fibroblasts derived from aged individuals by utilizing RNA-seq data from the public aging dataset GSE113957 (106). This dataset includes RNA sequences from primary skin fibroblast lines sourced from 143 donors, ranging in age from 2 months to 96 years. Gene expression clustering was performed to group genes based on age-related expression trends (Supplementary Table S7). Of the 12 clusters generated, three exhibited a decreasing expression pattern with advancing donor age (Supplementary Figure S6A). Specifically, cluster 2 showed an early decrease in expression, mainly representing genes associated with development (Supplementary Table S7). In contrast, cluster 3 demonstrated a gradual, continuous decline in expression with age, while cluster 7 showed a consistent decrease, with a pronounced reduction at advanced ages (Supplementary Figure S6A). Notably, clusters 3 and 7 were significantly enriched for DNA repair pathways, unlike cluster 2 (Supplementary Table S7).

**Figure 4.**
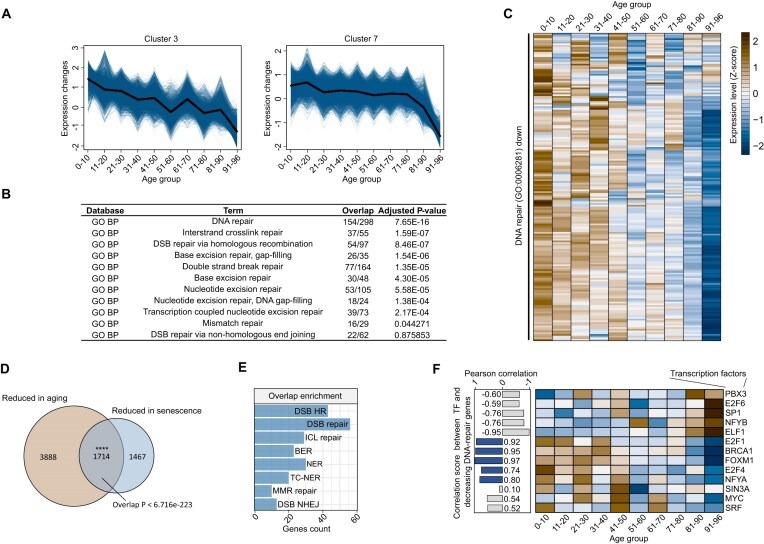
Shared DNA repair repression in senescent cells and proliferating fibroblasts from aging donors. (**A**) Gene expression clusters across donor age, illustrating decreasing expression patterns in fibroblast lines as donor age increases. Data sourced from GEO (accession number GSE113957). (**B**) Enriched DNA repair pathways identified after merging the two clusters from panel (A). (**C**) Heatmap showing downregulated DNA repair genes, with a marked decline in expression in cells derived from donors in their eighth decade of life. (**D**) Venn diagram depicting the overlap between downregulated genes during senescence (adjusted *P*-value <0.01, log_2_FC < −0.58 in the REM) and those downregulated in proliferating cells from aging individuals (adjusted *P*-value <0.01, log_2_FC < −0.58 in the ‘90–96’ age group compared to the ‘0–10’ age group). The overlap is statistically significant (*P*-value <0.0001, based on a hypergeometric test using all protein-coding genes as the background). (**E**) Enrichment plot showcasing DNA repair pathways associated with the overlapping genes identified in panel (D). (**F**) Heatmap of TF expression levels identified in Figure 2H. The bar chart on the left indicates correlation scores between TF expression and the median expression of DNA repair genes across different donor age groups.

Combining data from clusters 3 and 7, we observed the downregulation of numerous genes involved in major DNA repair pathways as donor age increased (Figure 4A and B). A heatmap of these DNA repair genes highlighted a significant reduction in expression, particularly evident starting in the eighth decade of life (Figure 4C). A comparison between these age-related downregulated genes and those observed in senescent cells revealed a significant overlap (representation factor: 1.8, *P* < 6.716E−223, based on a hypergeometric test using all protein-coding genes as the background). Over half of the genes downregulated in senescent cells were also downregulated with aging (Figure 4D), and this overlapping set showed strong enrichment for DNA repair pathways (Figure 4E) as well as other related processes (Supplementary Figure S6B). These findings indicate a marked decline in DNA repair capacity in late-stage human aging, mirroring patterns observed in cellular senescence.

We further analyzed the expression trends of TFs identified as potential upstream regulators of downregulated DNA repair genes in RS. The expression of these TF-encoding genes was visualized through a heatmap (Figure 4F, right panel) and correlated with DNA repair gene expression (Figure 4F, left panel). Notably, TFs such as E2F1, E2F4, FOXM1, BRCA1 and NFYA showed consistent declines in expression that correlated with reductions in DNA repair gene expression in both biological conditions. These results suggest that reduced expression of these TFs may contribute to the diminished DNA repair capacity observed during human aging.

## Discussion

In this study, we conducted a comprehensive meta-analysis using two distinct approaches: (i) a simple counting method that scored all genes based on their frequency of deregulation across the selected studies (Supplementary Table S2A) and (ii) a statistical integration of these studies into a ‘super-study’ using the REM tool to enhance statistical power (Supplementary Table S4). The first approach facilitated effective gene scoring, highlighting the significance and consistency of gene deregulation during senescence. By incorporating a wide range of datasets, this method enabled the identification of a greater number of deregulated genes compared to previous studies that have examined transcriptomic changes during senescence (Supplementary Table S2A and B).

A distinctive feature of our study is the comparative analysis between senescent and quiescent cells—two related but distinct cellular states (107). This analysis identified genes that are either commonly or uniquely deregulated in these conditions, shedding light on their shared and individual physiological properties (Supplementary Table S2A and B). The comprehensive gene list generated, in combination with the REM approach (Supplementary Table S4), offers significant potential for identifying novel senescence markers, key regulatory networks and new SASP candidates.

Our unique methodology provided an extensive overview of the complex transcriptome dynamics during senescence across various cell lines and in response to different senescence inducers. This analysis underscored a striking phenomenon previously documented in more limited contexts (33–36): the extensive downregulation of genes encoding key components of major DNA repair pathways in senescent cells. In cells undergoing RS, this downregulation becomes more pronounced with increasing population doublings, culminating in a significant decline as cells transition to overt senescence. The observation of this process in proliferating cell lines derived from aging donors suggests that it may also occur *in vivo* as tissues age.

The transcriptomic and proteomic phenomena we observed suggest a significant reduction in the activity of various DNA repair pathways, including NER, BER, NHEJ, HR, the FA pathway, MMR, RER and, at least partially, SSBR. Collectively, these pathways are essential for repairing continuous DNA damage, primarily induced by endogenous ROS (3,6). Although pathways such as RER, MMR and HR are typically associated with DNA replication—which is absent in senescent cells—they remain relevant. MMR is crucial for correcting mismatches arising from spontaneous deamination of DNA bases or during repairs involving error-prone DNA polymerases (108). HR may retain activity in senescent cells arrested in G2 (109), but this activity is likely impaired due to gene repression. Moreover, the gene encoding PARP1, a critical player in various DDR branches (DSB, NER and BER) (84), was found to be downregulated in 22 of the 27 datasets analyzed, and we observed a corresponding reduction in PARylation activity in senescent cells. This observation is consistent with previous reports of PARP1 repression in senescent epithelial cells (110). Our experimental findings supported the conclusions of our bioinformatic analysis, highlighting severe impairment of DNA repair capacity in senescent cells. However, the computational analysis presented in this study spans a broader range of cellular contexts and diverse senescence triggers.

**Figure 5.**
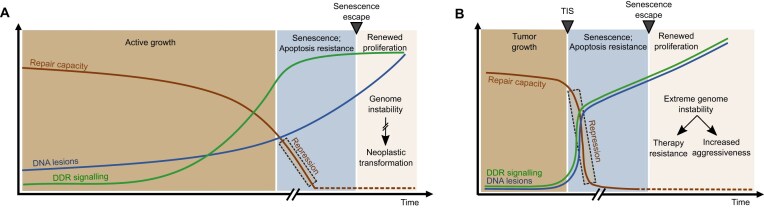
Predicted impact of DNA repair repression on senescence outcomes. (**A**) RS: During the proliferative phase (brown zone), DNA repair capacity gradually declines, leading to the steady accumulation of DNA lesions as cells approach senescence. Upon entry into the senescent state (blue zone), DNA repair capacity is markedly reduced repressed, resulting in further accumulation of DNA damage. Accumulating DNA lesions activate DDR signaling; however, the impaired repair pathways fail to effectively resolve the lesions. Despite the increasing DNA damage, senescent cells exhibit resistance to apoptosis, elevating the risk of genome instability. If these cells evade the senescent state, the associated genome instability can increase the likelihood for tumorigenesis (beige zone). (**B**) TIS: It involves a rapid onset of senescence, anticipated to cause a marked reduction in DNA repair capacity within senescent tumor cells. Should these cells escape senescence and replicate their highly damaged genome, the resulting elevated mutation rate and genome instability, combined with the malignant characteristics of the original cells, may drive tumor recurrence with increased aggressiveness and resistance to further therapies.

In our examination of data from primary cell lines undergoing RS, we observed a gradual decrease in DNA repair gene expression levels. This trend aligns with previous investigations of transcriptome dynamics during extended cultivation of various human (68,71,111) and mouse (59,112) primary cell lines. The progressive decline in DNA repair capacity likely contributes to the accumulation of DNA damage observed in these cell lines as they advance through successive passages until reaching RS (9). This phenomenon is thought to create a feedback loop that further propels cells toward a senescent state (33). This concept is consistent with the evolutionary theory of ‘antagonistic pleiotropy’ (113,114), which proposes that certain gene products provide short-term benefits but become detrimental over time. In the context of senescence, the initial suppression of DNA repair may facilitate a quicker transition into senescence and reduce energy expenditure. However, this repression could pose long-term risks, potentially increasing the susceptibility to tumorigenesis (Figure 5).

The phenomenon we describe appears to be a fundamental characteristic of senescent cells. Notably, it occurs in the context of apoptosis resistance and elevated metabolic activity, which is expected to generate substantial amounts of ROS (115), further exacerbating DNA damage. The suppression of the BER pathway, essential for repairing ROS-induced DNA lesions (79), could be particularly detrimental under these conditions. Consequently, the downregulation of DNA repair pathways in senescent cells is likely to result in the significant accumulation of DNA lesions. Furthermore, the reactivation of transposable elements—a well-documented occurrence during senescence (60,116)—introduces an additional layer of genomic instability.

The accumulation of DNA lesions is expected to compromise transcription fidelity, leading to ‘transcriptional mutagenesis’ (117–120), which may be further exacerbated by the decreased spliceosome expression we observed. This phenomenon has the potential to disrupt cellular functions randomly, potentially affecting the maintenance of the senescent state (29). However, the greatest threat to genome stability arises in cells that escape senescence and re-enter the cell cycle. As these cells replicate and propagate damaged DNA, such lesions can lead to a surge in sequence alterations and significant chromosomal instability. The accumulation of mutations and chromosomal aberrations over successive cell divisions may create a conducive environment for neoplastic transformation.

Senescent cells that can resist apoptosis and resume the cell cycle could thus be likened to a ‘ticking bomb’. This is especially relevant in the context of TIS, which has been linked to tumor recurrence, even after prolonged periods of dormancy (27–31). Relapsed tumors often exhibit greater aggressiveness than primary tumors, a trait driven largely by the accumulation of mutations and chromosomal abnormalities (29,121). Our study, along with others, indicates that this mutational burden can, at least in part, be attributed to the repression of DNA repair pathways during senescence. Moreover, genome instability has been proposed as a factor contributing to senescence escape (122).

Motif analysis of regulatory sequences within DNA repair genes that were downregulated during senescence revealed target sequences for a distinct set of TFs. Notably, these TFs have been previously implicated in the regulation of cell cycle dynamics and DNA repair processes (98–100,104,123). This finding suggests a coordinated downregulation of these critical processes during senescence (124). Additionally, these TFs were recently identified as potential drivers of senescence (123). However, that study reported only four DNA repair genes as downstream targets, all of which are G2-related processes.

The E2F family of TFs is well known for its critical role in regulating cell cycle progression and DNA repair (98) and for orchestrating cell cycle arrest during senescence (125,126). Among E2F members, E2F4 is particularly notable in the context of senescence (112). E2F1, a key regulator of DNA repair gene expression (98), is directly recruited to DSB sites (104). Previous studies have identified E2F1 and E2F4 as potential upstream factors involved in repressing DNA repair during senescence via p21, though this effect has been primarily observed in G2-related pathways, such as MMR and HRR (35,36). NFYA, a TF that facilitates cell cycle progression during the G1 phase (127), also plays a role in arresting cellular proliferation during senescence (128–130) and modulates DNA repair gene expression (131). We observed a marked decrease in *FOXM1* expression at the onset of senescence. FOXM1, which is stabilized in response to DNA damage, is known to activate genes involved in the BER, NER, NHEJ and HRR pathways (101–103). Reduced levels of FOXM1 have been associated with aging, genome instability and mitotic defects (90). Notably, FOXM1 can convert the DREAM complex from a repressor to an activator (105). Given that the DREAM complex represses DNA repair genes (86), the downregulation of FOXM1 during senescence may enhance DREAM-mediated repression of DNA repair pathways. These findings highlight the potential of FOXM1 as a therapeutic target in progeria and other aging-related diseases (91). Future studies should investigate the causal relationship between senescence and DNA repair repression. Manipulating senescence-inducing factors, such as p21 and p16, and evaluating DNA repair efficiency could provide valuable insights. Additionally, investigating the role of TFs, such as FOXM1, as well as chromatin modifications, could shed light on the underlying mechanisms regulating DNA repair during senescence. Genetic manipulation and techniques such as Chip-seq (chromatin immunoprecipitation sequencing, ATAC-seq (assay for transposase-accessible chromatin using sequencing) and omics approaches could be employed to identify key regulators and their targets.

The gradual decline in DNA repair observed in proliferating cell lines derived from older donors aligns with previous reports of increased DNA damage in tissue cells from aging individuals (9,132,133). Notably, we found that many DNA repair genes downregulated in cell lines from older donors are also repressed during cellular senescence, suggesting a link between the mechanisms underlying aging and senescence. The repression of DNA repair in aging skin fibroblasts may result from gradual accumulation of senescent cells or the indirect influence of the SASP phenotype affecting neighboring cells. The age-related decline in genomic maintenance could be attributed to the absence of strong selective pressures for maintaining genome integrity in older individuals throughout most of human history, as natural selection may not have favored the preservation of these mechanisms in later life. Furthermore, the accumulation of senescent cells in the tissues of elderly individuals (23,26) further underscores the relationship between aging and senescence. A more direct link between DNA repair capacity and aging is evident from the segmental premature aging observed in various human genome instability disorders (134,135) and in mouse models deficient in DNA repair pathways, which exhibit signs of premature aging and accelerated tissue senescence (136–138).

In conclusion, our study provides a novel, high-resolution perspective on transcriptomic dynamics during cellular senescence. This approach has revealed a critical aspect of these dynamics: the repression of DNA repair pathways during senescence. This suppression likely has significant effects on the phenotype and fate of cells that re-enter the cell cycle after senescence, carrying important clinical implications for tumor recurrence driven by escape from TIS.

## Supplementary Material

gkae1257_Supplemental_Files

## Data Availability

The raw data underlying this article are publicly available and can be accessed through the Gene Expression Omnibus (https://www.ncbi.nlm.nih.gov/geo/) and ArrayExpress (https://www.ebi.ac.uk/biostudies/arrayexpress) databases. Detailed information about the data, including accession IDs, related publications and authors, is provided in Supplementary Table S1. The results of the data analysis, along with the supplementary tables, are included in the article and its online supplementary material.
