# Correction to ‘Broad repression of DNA repair genes in senescent cells identified by integration of transcriptomic data’

**DOI:** 10.1093/nar/gkaf430

**Published:** 2025-05-14

This is a correction to: Yann Frey, Majd Haj, Yael Ziv, Ran Elkon, Yosef Shiloh, Broad repression of DNA repair genes in senescent cells identified by integration of transcriptomic data, *Nucleic Acids Research*, Volume 53, Issue 1, 13 January 2025, gkae1257, https://doi.org/10.1093/nar/gkae1257

The authors have substituted the PDF versions of Supplementary Tables 2A, 2B and 3B with Excel files to facilitate navigation.

This change does not affect the results, discussion and conclusions presented in the article. The published article has been updated.

