# Supplementary material for: Broad repression of DNA repair genes in senescent cells identified by integration of transcriptomic data
Source: Nucleic Acids Res. 2024 Dec 31;53(1):gkae1257. doi: 10.1093/nar/gkae1257 (PMC11724277; doi:10.1093/nar/gkae1257)
Supplement: gkae1257_Supplemental_Files [file gkae1257_supplemental_files.zip › Supplementary_information_F.pdf]

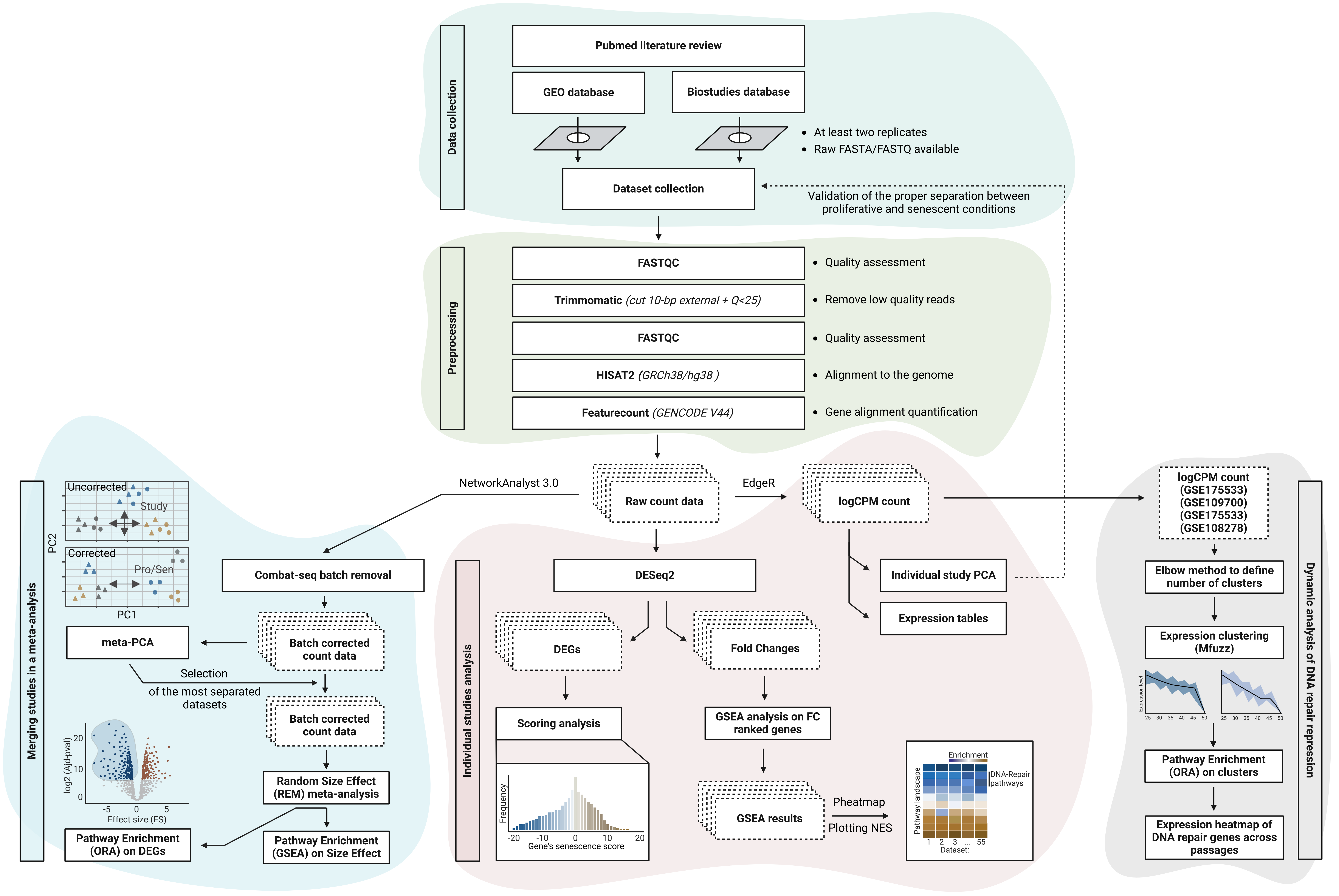
Data collection
Pubmed literature review
GEO database
Biostudies database
At least two replicates
Raw FASTA/FASTQ available
Dataset collection
Validation of the proper separation between proliferative and senescent conditions
Preprocessing
FASTQC
Quality assessment
Trimmomatic (cut 10-bp external + Q<25)
Remove low quality reads
FASTQC
Quality assessment
HISAT2 (GRCh38/hg38)
Alignment to the genome
Featurecount (GENCODE V44)
Gene alignment quantification
NetworkAnalyst 3.0
Raw count data
EdgeR
logCPM count
logCPM count (GSE175533) (GSE109700) (GSE175533) (GSE108278)
Merging studies in a meta-analysis
Uncorrected
Study
Corrected
Pro/Sen
PC2
PC1
Combat-seq batch removal
meta-PCA
Batch corrected count data
Selection of the most separated datasets
Batch corrected count data
log2 (Ajd-pval)
Effect size (ES)
Random Size Effect (REM) meta-analysis
Pathway Enrichment (ORA) on DEGs
Pathway Enrichment (GSEA) on Size Effect
Individual studies analysis
DESeq2
Individual study PCA
Expression tables
DEGs
Fold Changes
Scoring analysis
Frequency
Gene's senescence score
GSEA analysis on FC ranked genes
GSEA results
Pheatmap
Plotting NES
Enrichment
DNA-Repair pathways
Pathway landscape
Dataset:
Dynamic analysis of DNA repair repression
Elbow method to define number of clusters
Expression clustering (Mfuzz)
Expression level
Pathway Enrichment (ORA) on clusters
Expression heatmap of DNA repair genes across passages

Supplementary Figure 1. A comprehensive flowchart illustrating the analysis pipeline, with further details provided in the Materials and Methods section. The pipeline comprises five main stages: (1) Data selection and collection, involving the identification and acquisition of relevant datasets; (2) Preprocessing of raw data; (3) Independent analysis of individual studies; (4) Meta-analysis of merged datasets; and (5) Dynamic analysis of DNA repair repression through expression clustering. Notably, the dynamic analysis model was specifically employed for the aging analysis.

Frey et al. Supplementary Figure 2

A

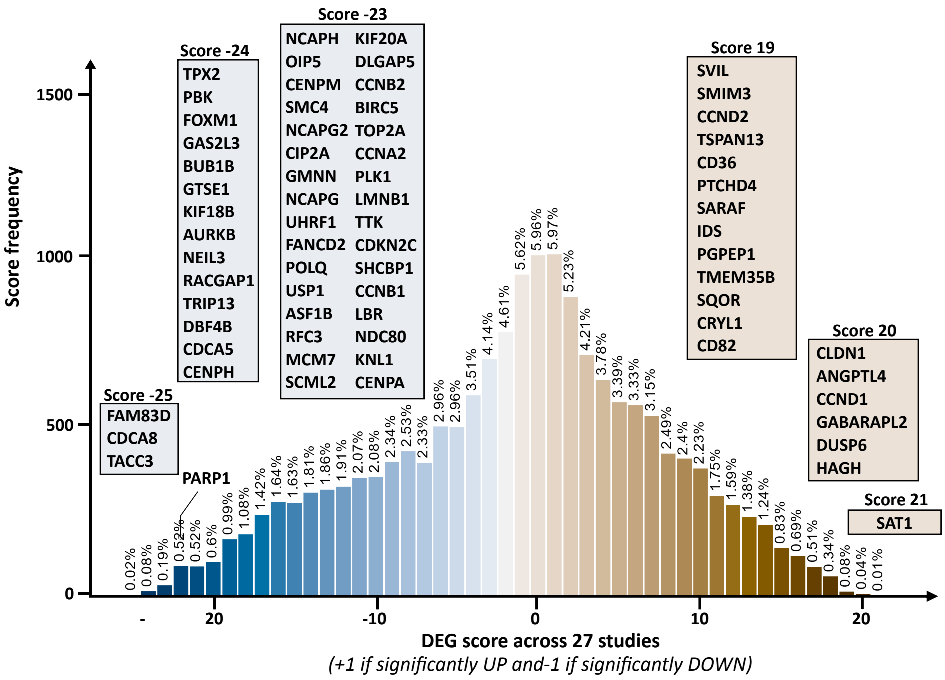

Frey et al. Supplementary Figure 2 (Con't #1)

B

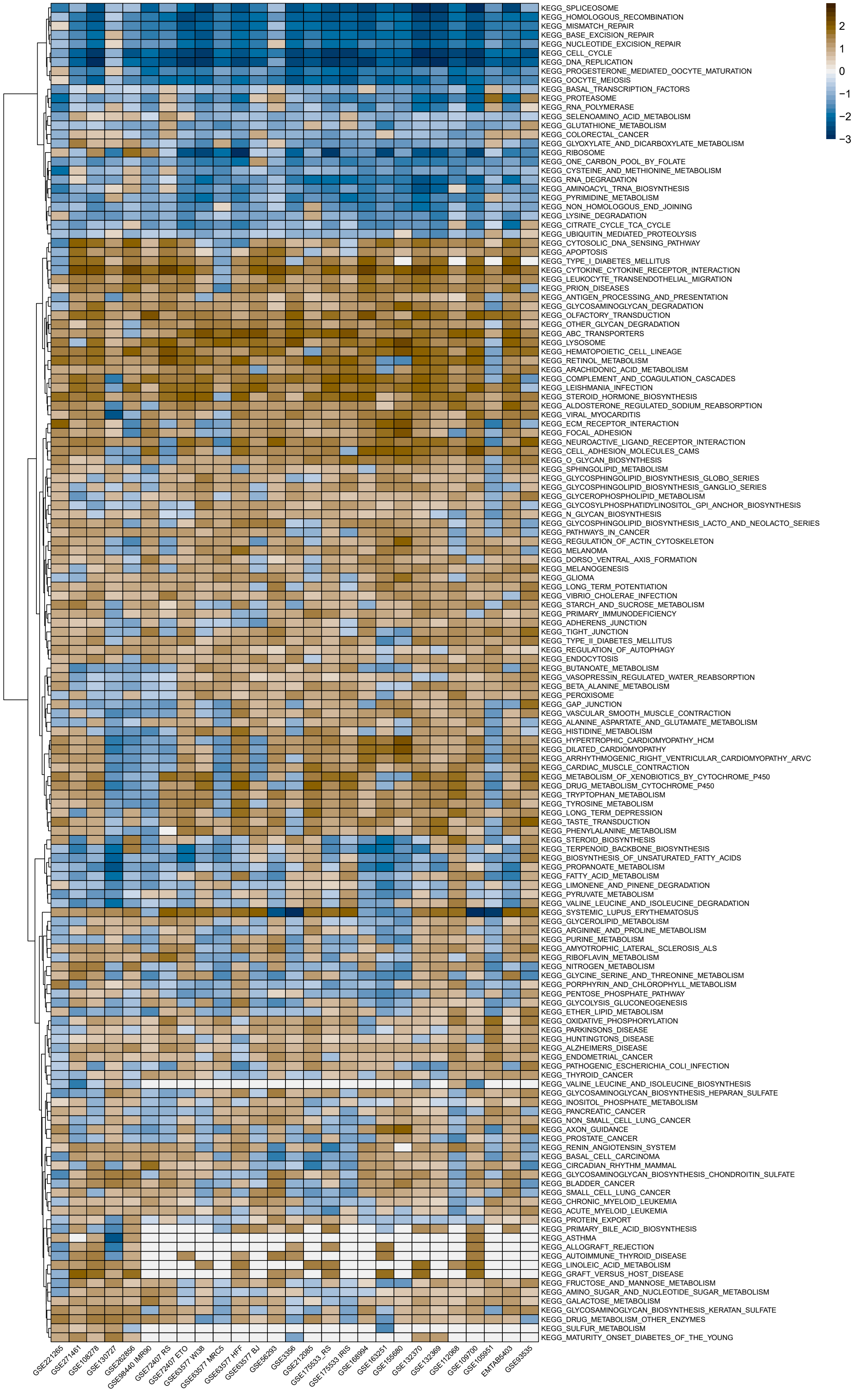

Frey et al. Supplementary Figure 2 (CON'T #2)

C

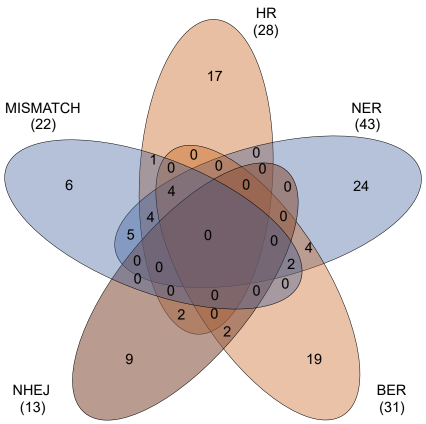

D

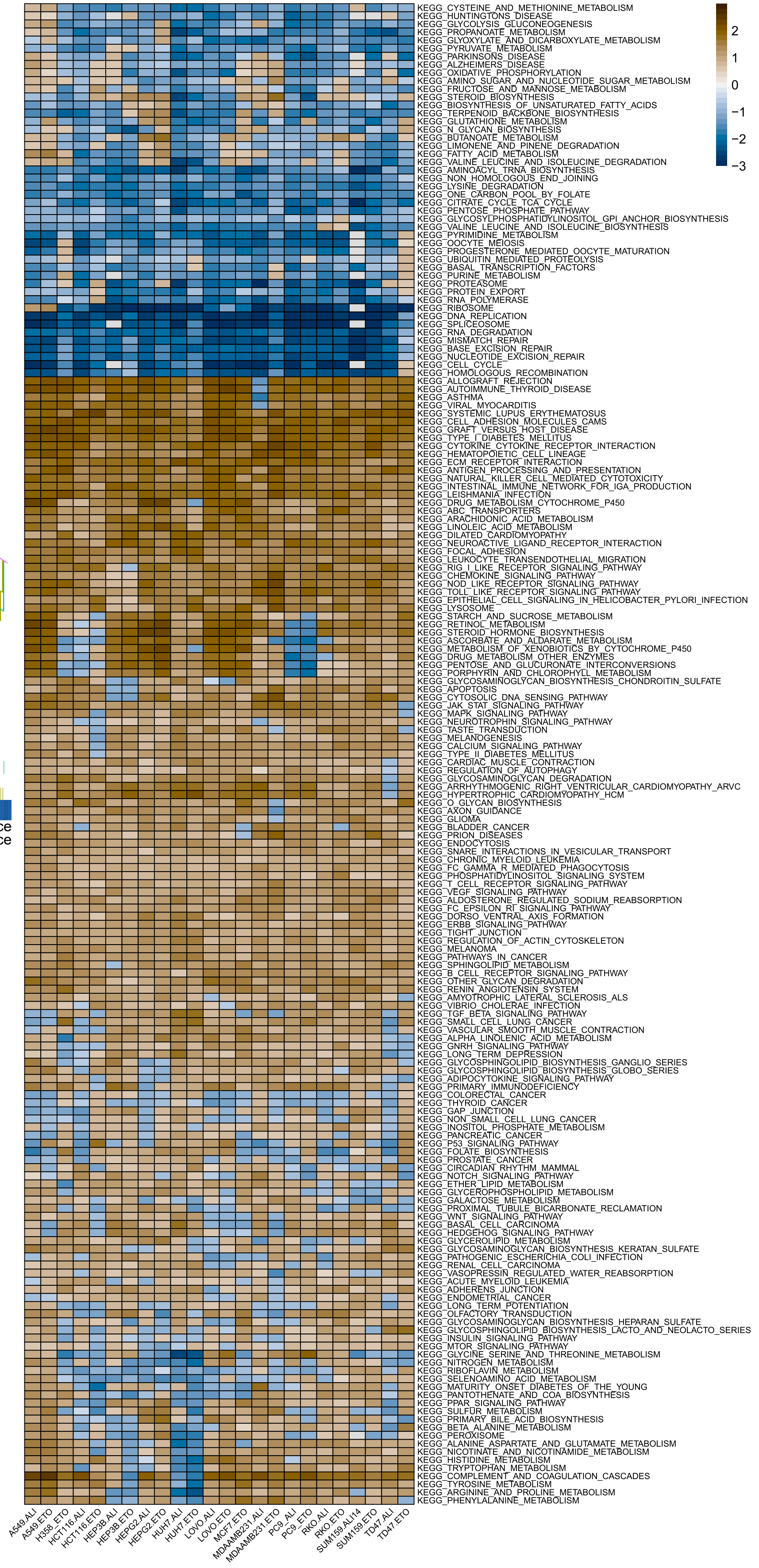

E

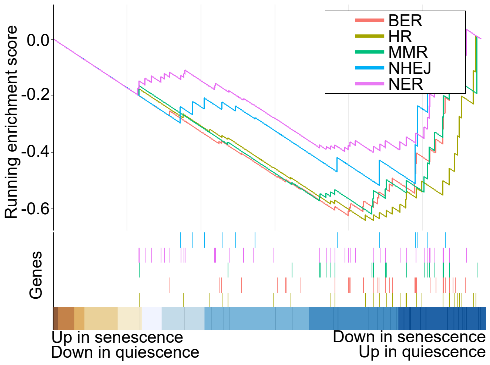

Supplementary Figure 2. (A) Distribution curve illustrating the scores from the senescence meta-analysis of 25 studies focusing on primary cell lines. Genes were assigned scores with (+1) for up-regulation and (-1) for down-regulation in each study, resulting in a distribution where consistently up-regulated genes are represented on the right tail and down-regulated genes - on the left tail. **(B)** Heatmap showing GSEA scores (normalized enrichment scores, NES) for KEGG pathways comparing proliferative and senescent cells. Unlike Figure 1A, this heatmap includes all KEGG pathways, even those without significant changes in behavior. **(C)** Venn diagram illustrating DNA repair pathways associated with genes that are down-regulated in senescence. **(D)** Comprehensive heatmap displaying GSEA scores (NES) for significantly enriched KEGG pathways across 25 datasets comparing proliferative vs. senescent cells in cell undergoing senescence induced by chemotherapeutic agents. Data was obtained from the SENESCopedia database, providing an extensive overview of KEGG pathways. **(E)** GSEA plot illustrating the down-regulation of DNA repair pathways in senescence relative to quiescence (GEO = GSE93535). Reduced expression of DNA repair genes is notably specific to senescence. Adjusted p-values for all plotted pathways are less than 0.01, except for NHEJ. The color scale indicates expression levels, from brown (up-regulated) to blue (down-regulated).

Frey et al. Supplementary Figure 3

A

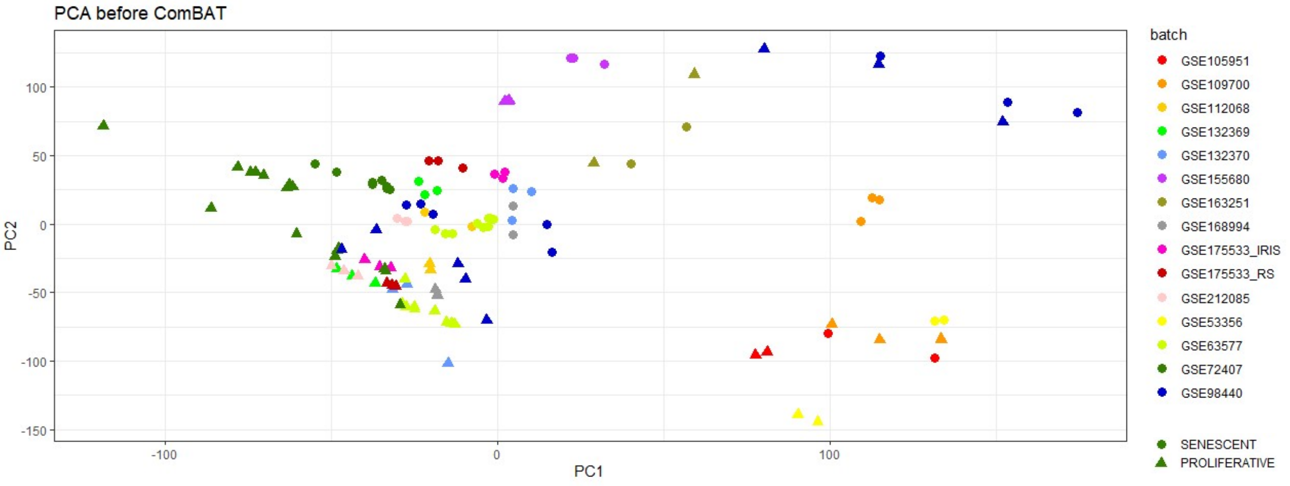

B

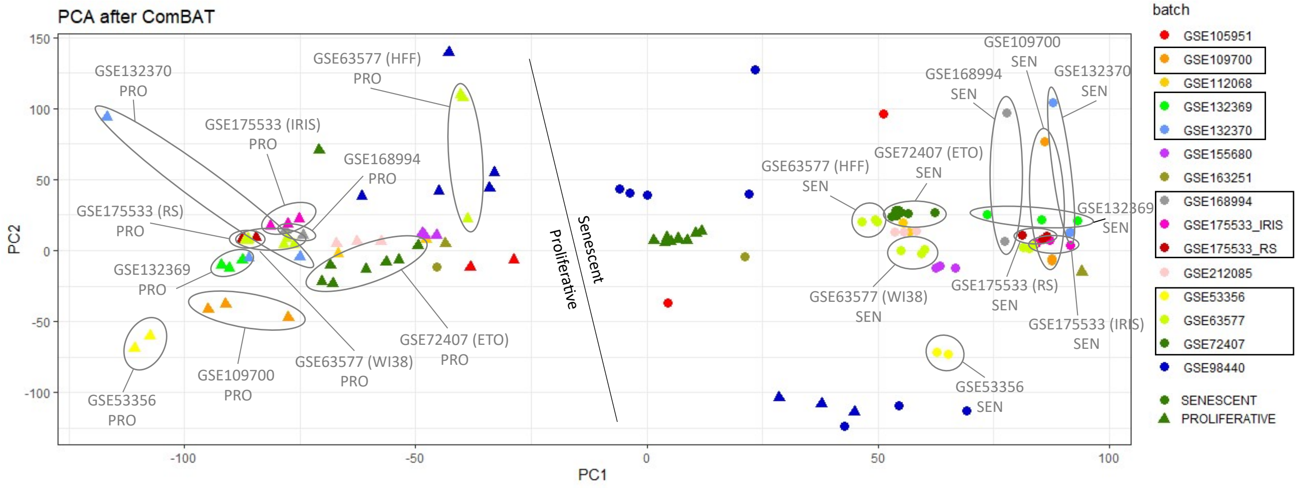

C

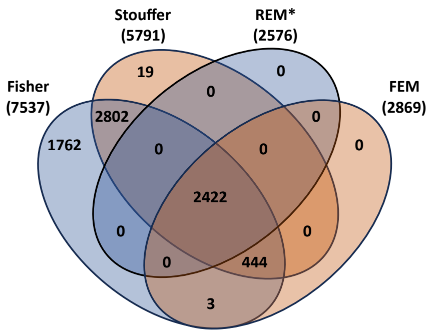

Supplementary Figure 3. (**A**) Principal component analysis (PCA) performed prior to batch correction shows that sample separation is primarily influenced by their origin rather than their cellular state (proliferative or senescent). This separation is attributed to batch effects arising from differences in sequencing protocols and laboratory conditions. (**B**) PCA analysis post-batch correction reveals successful separation of samples based on their cellular state, distinguishing between senescent and proliferative cells. Light gray circles indicate the selected samples chosen for data merging and subsequent meta-analysis, with corresponding accession numbers and cellular states specified. (**C**) Venn diagram illustrating genes significantly down-regulated in senescent cells as identified by various meta-analysis methods. A total of 2,422 genes were found to be down-regulated across the four different methods. The Random Effects Model (REM), known for its stringent criteria, was selected for further analysis.

Frey et al. Supplementary Figure 4

A

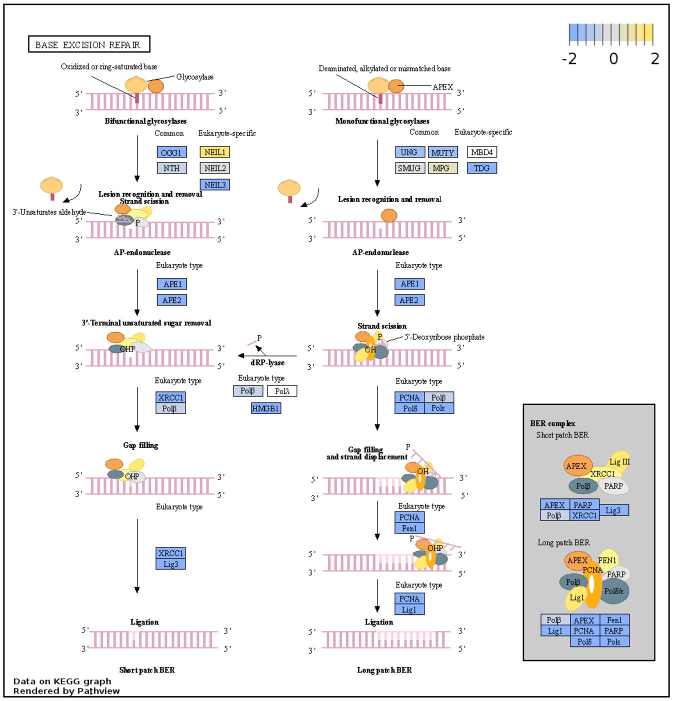

B

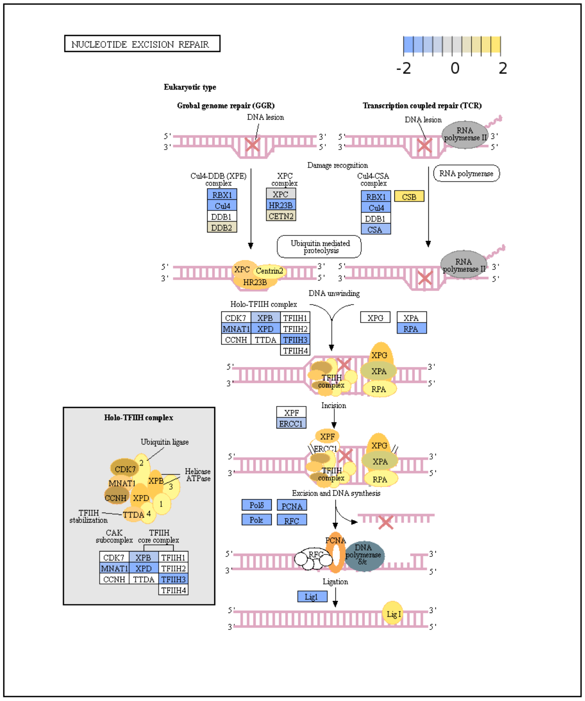

C

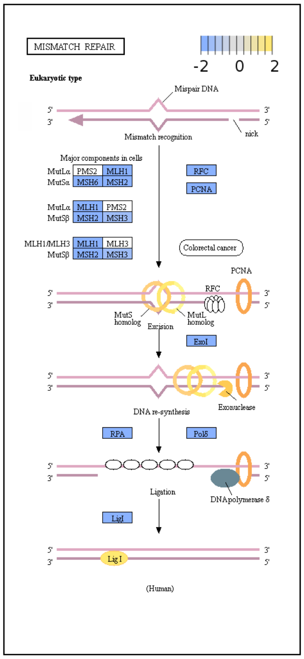

D

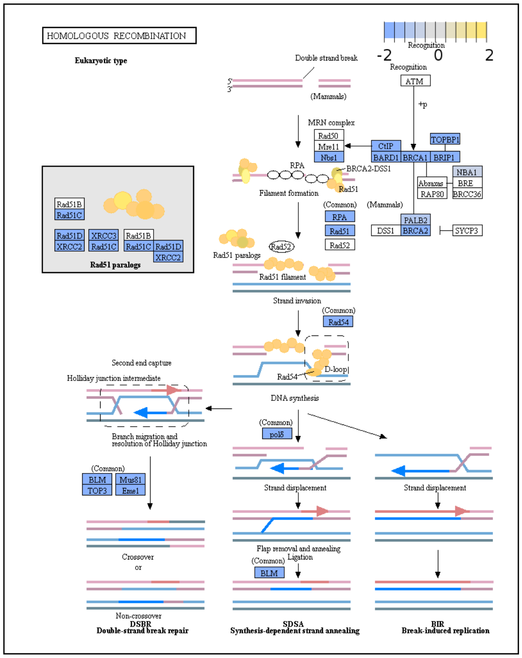

E

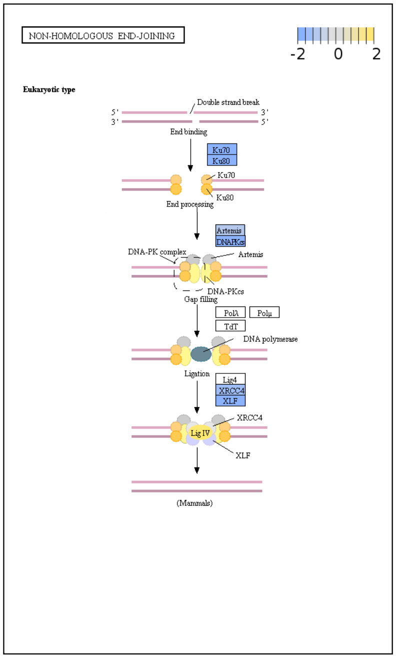

Frey et al. Supplementary Figure 4 (CONT)

F

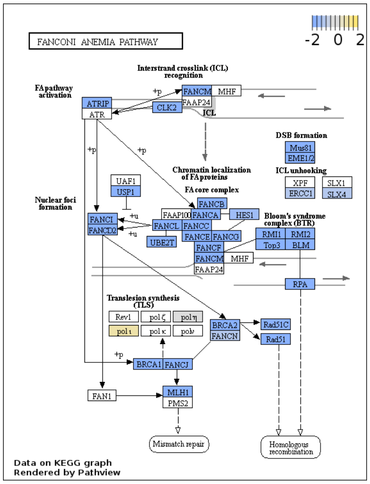

G

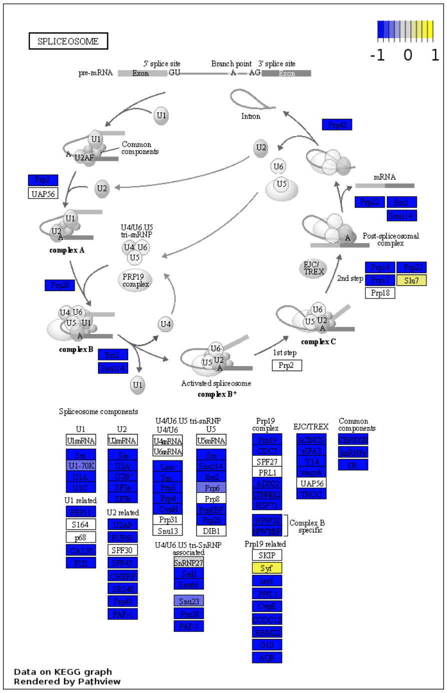

Supplementary Figure 4. Pathview plots illustrating the DNA repair and RNA splicing pathways. Proteins within these pathways are color-coded according to the expression fold-change of their genes, with yellow indicating up-regulated expression and blue representing down-regulated expression. Note that for clarity, sections specific to prokaryotes and *Saccharomyces cerevisiae* were removed from the plots.

Frey et al. Supplementary Figure 5

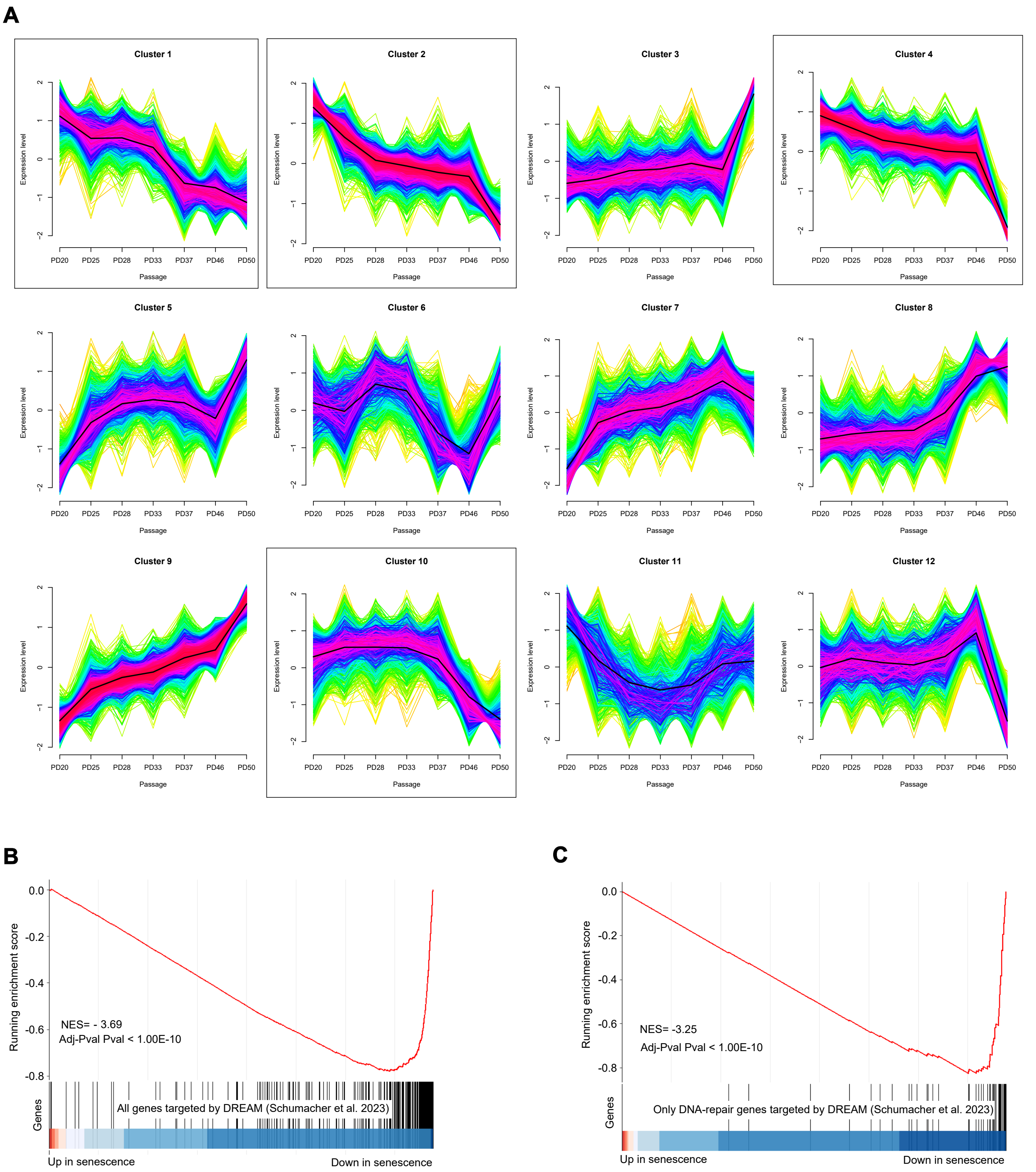

Frey et al. Supplementary Figure 5

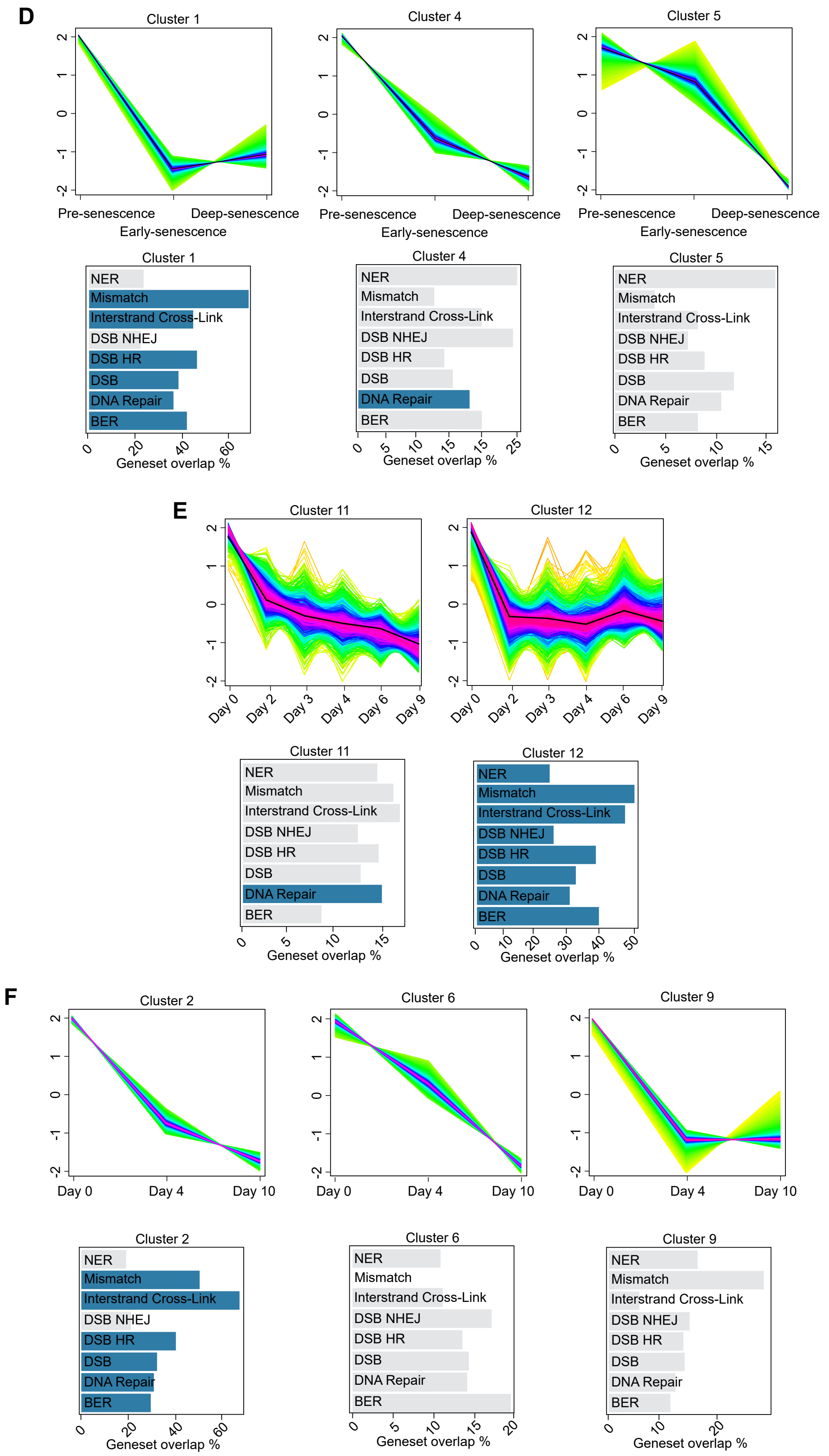

Supplementary Figure 5. (A) Expression clusters identified through Mfuzz analysis in the replicative senescence dataset, GSE175533. The X-axis represents the passage level, while the Y-axis represents the expression level. The down-regulated clusters selected for enrichment analysis in Figure 3B are highlighted within black boxes. **(B)** GSEA plot demonstrating the down-regulation of DREAM targets, as defined by Bujarrabal-Dueso et al 2023 (ref. 87) **(C)** GSEA plot demonstrating the down-regulation of DNA repair genes targeted by DREAM, as defined in Bujarrabal-Dueso et al 2023 (ref. 87) **(D)** Expression clusters identified in the IMR90 replicative senescence dataset, GSE109700. The X-axis represents the cellular state, while the Y-axis represents the expression level. Only downregulated pathways are displayed. The right panel present the corresponding enrichment results for each cluster. Only DNA repair pathways are displayed. Significant pathways are highlighted in blue (Adj P-value < 0.05). **(E)** Expression clusters identified in the WI38 irradiation induced senescence dataset, GSE175533. The X-axis represents the passage level, while the Y-axis represents the expression level. Only downregulated pathways are displayed. The right panel present the corresponding enrichment results for each cluster. Only DNA repair pathways are displayed. Significant pathways are highlighted in blue (Adj P-value < 0.05). **(F)** Expression clusters identified in the IMR90 oncogene induced senescence dataset, GSE108278. The X-axis represents the day post oncogene induction, while the Y-axis represents the expression level. Only downregulated pathways are displayed. The right panel present the corresponding enrichment results for each cluster. Only DNA repair pathways are displayed. Significant pathways are highlighted in blue (Adj P-value < 0.05).

Frey et al. Supplementary Figure 6

A

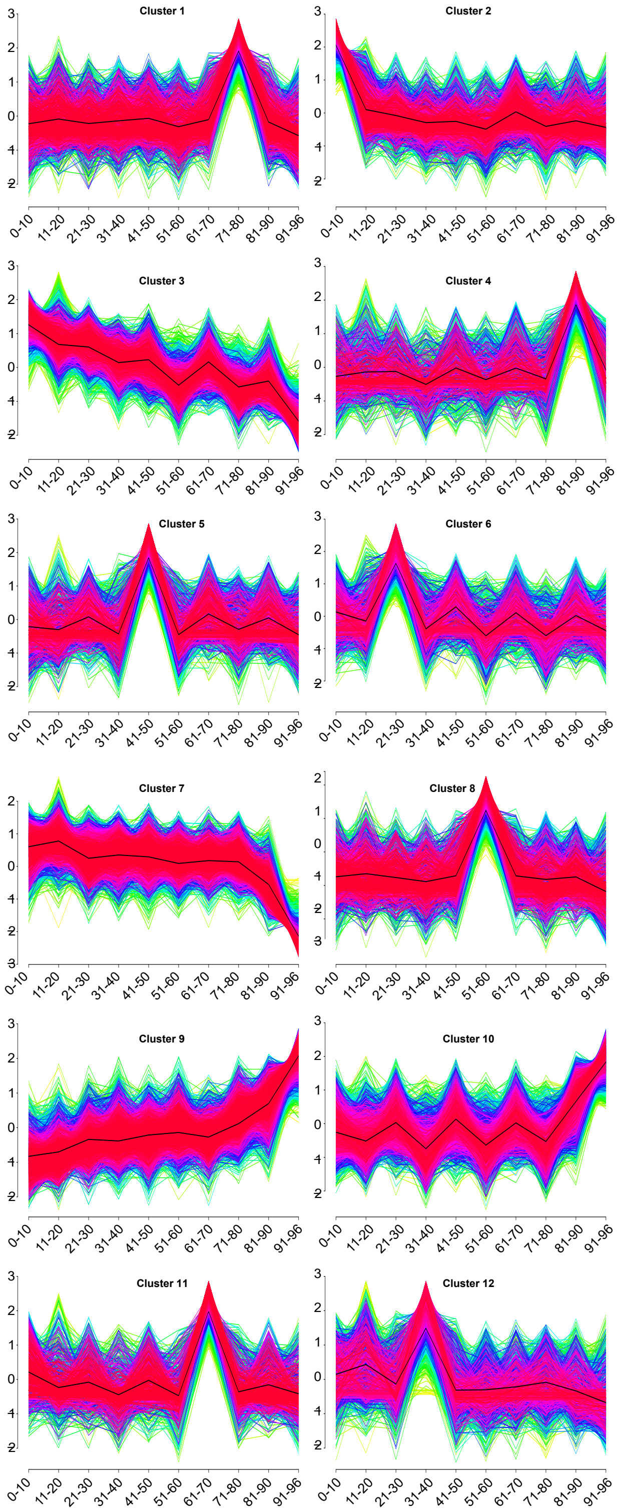

B

Term	Overlap	**Adjusted P-value**
Ribosome	77/158	9.39E-38
DNA replication	27/36	8.13E-20
Cell cycle	49/124	4.40E-19
Spliceosome	54/150	5.55E-19
RNA transport	56/186	1.13E-15
Fanconi anemia pathway	27/54	1.22E-13
Coronavirus disease	59/232	5.78E-13
Homologous recombination	22/41	5.27E-12
Base excision repair	17/33	6.65E-09
Proteasome	18/46	4.49E-07
Amyotrophic lateral sclerosis	64/364	6.24E-07
Mismatch repair	12/23	2.01E-06
Ribosome biogenesis in eukaryotes	26/108	2.28E-05
Oocyte meiosis	29/129	2.38E-05
Nucleotide excision repair	15/47	9.61E-05
Spinocerebellar ataxia	29/143	1.82E-04
RNA degradation	19/79	4.88E-04
Progesterone-mediated oocyte maturation	22/100	5.00E-04
Pyrimidine metabolism	15/56	7.85E-04

Suppl. Figure 6: (A) Expression clusters generated using Mfuzz analysis from the human aging dataset, GSE113957. The X-axis denotes the age groups, while the Y-axis indicates expression level. Clusters showing down-regulated expression, which were selected for enrichment analysis in Fig. 4A, are highlighted with black boxes. **(B)** Top-enriched pathways identified for the subset of overlapping gene that are down-regulated in both human aging and senescence, as shown in Fig. 4D.
